# α-1,3-Glucanase from the gram-negative bacterium *Flavobacterium* sp. EK-14 hydrolyzes fungal cell wall α-1,3-glucan

**DOI:** 10.1038/s41598-023-48627-y

**Published:** 2023-12-05

**Authors:** Masaki Takahashi, Shigekazu Yano, Yui Horaguchi, Yuitsu Otsuka, Wasana Suyotha, Koki Makabe, Hiroyuki Konno, Susumu Kokeguchi

**Affiliations:** 1 Graduate School of Sciences and Engineering, Yamagata University, Jonan, Yonezawa, Yamagata 992-8510 Japan; 2 Enzyme Technology Laboratory, Faculty of Agro-Industry, Prince of Songkla University, Hat Yai, 90112 Thailand; 3 Department of Oral Microbiology, Graduate School of Medicine, Dentistry and Pharmaceutical Sciences, Okayama University, Okayama, 700-8525 Japan

**Keywords:** Enzymes, Glycobiology

## Abstract

The glycoside hydrolase (GH) 87 α-1,3-glucanase (Agl-EK14) gene was cloned from the genomic DNA of the gram-negative bacterium *Flavobacterium* sp. EK14. The gene consisted of 2940 nucleotides and encoded 980 amino acid residues. The deduced amino acid sequence of Agl-EK14 included a signal peptide, a catalytic domain, a first immunoglobulin-like domain, a second immunoglobulin-like domain, a ricin B-like lectin domain, and a carboxyl-terminal domain (CTD) involved in extracellular secretion. Phylogenetic analysis of the catalytic domain of GH87 enzymes suggested that Agl-EK14 is distinct from known clusters, such as clusters composed of α-1,3-glucanases from bacilli and mycodextranases from actinomycetes. Agl-EK14 without the signal peptide and CTD hydrolyzed α-1,3-glucan, and the reaction residues from 1 and 2% substrates were almost negligible after 1440 min reaction. Agl-EK14 hydrolyzed the cell wall preparation of *Aspergillus oryzae* and released glucose, nigerose, and nigero-triose from the cell wall preparation. After treatment of *A. oryzae* live mycelia with Agl-EK14 (at least 0.5 nmol/ml), mycelia were no longer stained by red fluorescent protein-fused α-1,3-glucan binding domains of α-1,3-glucanase Agl-KA from *Bacillus circulans* KA-304. Results suggested that Agl-EK14 can be applied to a fungal cell wall lytic enzyme.

## Introduction

α-1,3-Glucan is an insoluble polysaccharide composed of glucose linked by α-1,3-glycosidic bonds. The glucan exists primarily as an extracellular polysaccharide produced by oral *Streptococcus*^1,2^. The polysaccharide of oral *Streptococcus* is synthesized by glycosyltransferases (GTFs) from sucrose^3,4^. This substance is highly adhesive, and oral *Streptococcus* uses its adhesive properties to adhere to tooth surfaces and form dental plaques^5^. Therefore, α-1,3-glucan-degrading enzymes (α-1,3-glucanases; EC 3.2.1.59) have been studied as potent enzymes for removing oral plaques.

α-1,3-Glucan also exists as a cell wall component in some fungi^6–9^. The fungal cell wall maintains the cellular structure and protects the cell from the external environment^10^. Recent studies have revealed that some pathogenic fungi rely on α-1,3-glucan to protect their cells from the host’s immune system. A human pathogenic fungus, *Histoplasma capsulatum*^11^, and a plant pathogenic fungus, *Magnaporthe oryzae*^12^, have been studied for their defense mechanisms using α-1,3-glucan. The genus *Aspergillus*, important in the fermentation industry, has been observed to accumulate α-1,3-glucan in the cell wall surface layer^8^. In the genetic modification-based molecular breeding of fungi, protoplasts are prepared from mycelia using cell wall lytic enzymes^13,14^. However, α-1,3-glucan in the *Aspergillus* cell wall interferes with cell wall lytic enzymes, inhibiting protoplast formation^15^. Therefore, there is a rising need for developing α-1,3-glucanase that can hydrolyze fungal cell wall α-1,3-glucan^16^.

α-1,3-Glucanases are categorized into two families—glycoside hydrolase (GH) 71 and 87—based on their amino acid sequences in the Carbohydrate-Active enZymes (CAZy) database (http://www.cazy.org)^17^. GH71 enzymes were found in fungi such as *Trichoderma*, *Penicillium*, *Aspergillus*, and *Schizosaccharomyces*^18–20^. GH87 enzymes were mainly found in gram-positive bacteria such as *Bacillus*, *Paenibacillus*, and *Streptomyces*^21–23^. Some GH87 enzymes from gram-positive bacteria were characterized, and X-ray crystallographic analysis of the catalytic domain was performed (e.g., Agl-KA of *Bacillus circulans* KA-304, Agl-FH1 of *Paenibacillus glycanilyticus* FH11, and Agl-ST of *Streptomyces thermodiastaticus* HF3-3)^24–26^. For gram-negative bacteria, α-1,3-glucanases have been isolated from *Flavobacterium*, *Pseudomonas*, *Bacteroides orali*, and *Paracoccus mutanolyticus*^27–30^. However, the α-1,3-glucanase genes have never been cloned.

This study investigated the cloning and expression of the gene encoding α-1,3-glucanase (Agl-EK14) in *Flavobacterium* sp. EK-14. In 1975, *Flavobacterium* sp. EK-14 was isolated from soil on a medium containing insoluble sticky glucan produced by *Streptococcus mutans* OMZ 176^27^. α-1,3-Glucanase (EK-14 enzyme) was purified from a culture filtrate of strain EK-14, and various properties of the enzyme were investigated. Furthermore, the EK-14 enzyme hydrolyzed α-1,3-glucans synthesized by oral *Streptococcus* as well as the glucan in fungal cell walls. These findings suggest that *Flavobacterium* is a potential source of α-1,3-glucanase genes. Here, an α-1,3-glucanase gene was cloned from strain EK-14 based on putative GH87 enzyme α-1,3-glucanase genes of *Flavobacterium* registered in the database. Agl-EK14 was also expressed in *Escherichia coli*, and the purified enzyme strongly hydrolyzed the α-1,3-glucan prepared by GTF-I of *S. mutans*. Agl-EK14 also hydrolyzed the cell wall α-1,3-glucan of *Aspergillus oryzae*, suggesting that GH87 α-1,3-glucanase from gram-negative bacteria is a useful enzyme for fungal control, as are GH87 enzymes from gram-positive bacteria and GH71 enzymes from fungi.

## Results

### Sequence analysis of the α-1,3-glucanase gene of *Flavobacterium* sp. EK-14

*Flavobacterium* is a gram-negative bacterium belonging to Bacteroidetes and is often isolated from soil and environmental water. α-1,3-Glucanase genes have never been cloned from *Flavobacterium* species. Therefore, this study cloned α-1,3-glucanase from strain EK-14 based on putative α-1,3-glucanases from the CAZy database. To amplify the gene via polymerase chain reaction (PCR), sense and antisense primers were designed based on this putative enzyme’s conserved region, such as *Flavobacterium anhuiense* M168 and *Flavobacterium johnsoniae* UW101. PCR product, approximately 2700-bp long, showed a high sequence similar to the α-1,3-glucanase encoding gene from *F. anhuiense* M168. The DNA fragment containing upstream and downstream regions of the partial Agl-EK14 gene was amplified by PCR, and the primers were designed based on the upstream and downstream regions of the open reading frame (ORF) of the *F. anhuiense* gene. As a result, an approximately 3000-bp PCR product was obtained. The product contains an ORF corresponding to about 980 amino acid residues (Supplementary Fig. S1), with a sequence highly similar to those of the putative enzymes described above (Supplementary Fig. S2), indicating that a complete α-1,3-glucanase gene was obtained.

**Figure 1:**
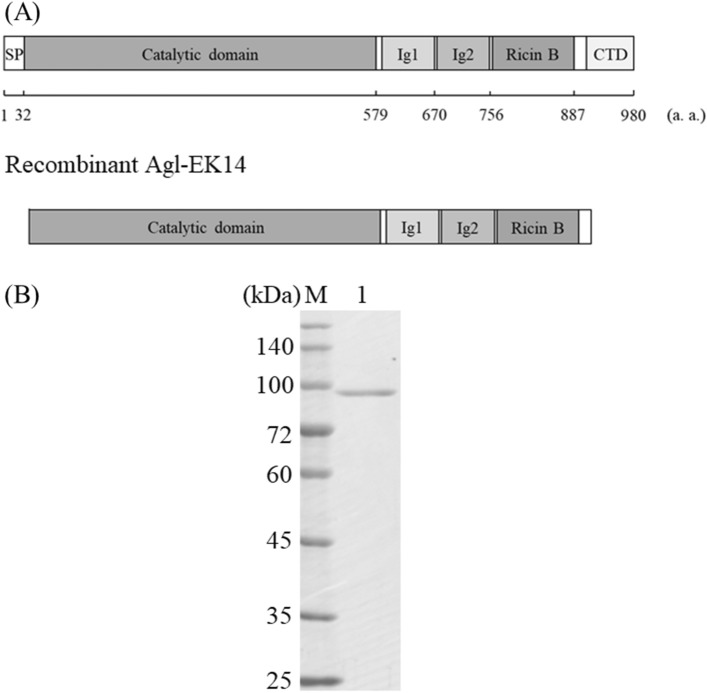
Schematic design of the domain of Agl-EK14 (**A**) and SDS-PAGE analysis of the purified proteins (**B**). (**A**) *SP* signal peptide, *Ig1* first immunoglobulin-like domain, *Ig2* second immunoglobulin-like domain, *Ricin B* Ricin B-like lectin domain, *CTD* carboxy-terminal domain. (**B**) The 10% SDS-PAGE gel was stained with Coomassie brilliant blue G250. Lane M, molecular weight markers; lane 1, Agl-EK14. The original image is presented in Supplementary Fig. S9.

To clarify the domain structure of α-1,3-glucanase, a homology search was performed against the InterPro database (http://www.ebi.ac.uk/interpro/). Figure 1A shows that the α-1,3-glucanase of *Flavobacterium* sp. EK-14 contains an amino-terminal signal peptide (SP) at Met1-Tyr32, a GH87 catalytic domain at Ala33-Gly579, a first immunoglobulin-like domain (Ig1) at Val589-Ser670, a second immunoglobulin-like domain at Val675-Ala756 (Ig2), a ricin B-like lectin domain (Ricin B) at Tyr761-Thr887, and a carboxyl-terminal domain (CTD) at Ile907-Lys980. Ig1, Ig2, Ricin B, and CTD sequences were not substantially similar to any characterized domains of known α-1,3-glucanases. The amino acid sequences of Ig1 and Ig2 had approximately 50% similarity (Supplementary Fig. S3A). Ig-like domains have been found in several GHs and reported to have various functions, such as increasing stability and binding to a substrate^31–33^. Ricin B is found in glycosidic hydrolytic enzymes involved in substrate binding, such as exo-β-1,3-galactanase and xylanase^34,35^. Ricin B of Agl-EK14 had low sequence similarity to known domains (Supplementary Fig. S3B). CTD has a role in the extracellular secretion of *Flavobacterium* enzymes and is cleaved during secretion^36,37^.

**Figure 2:**
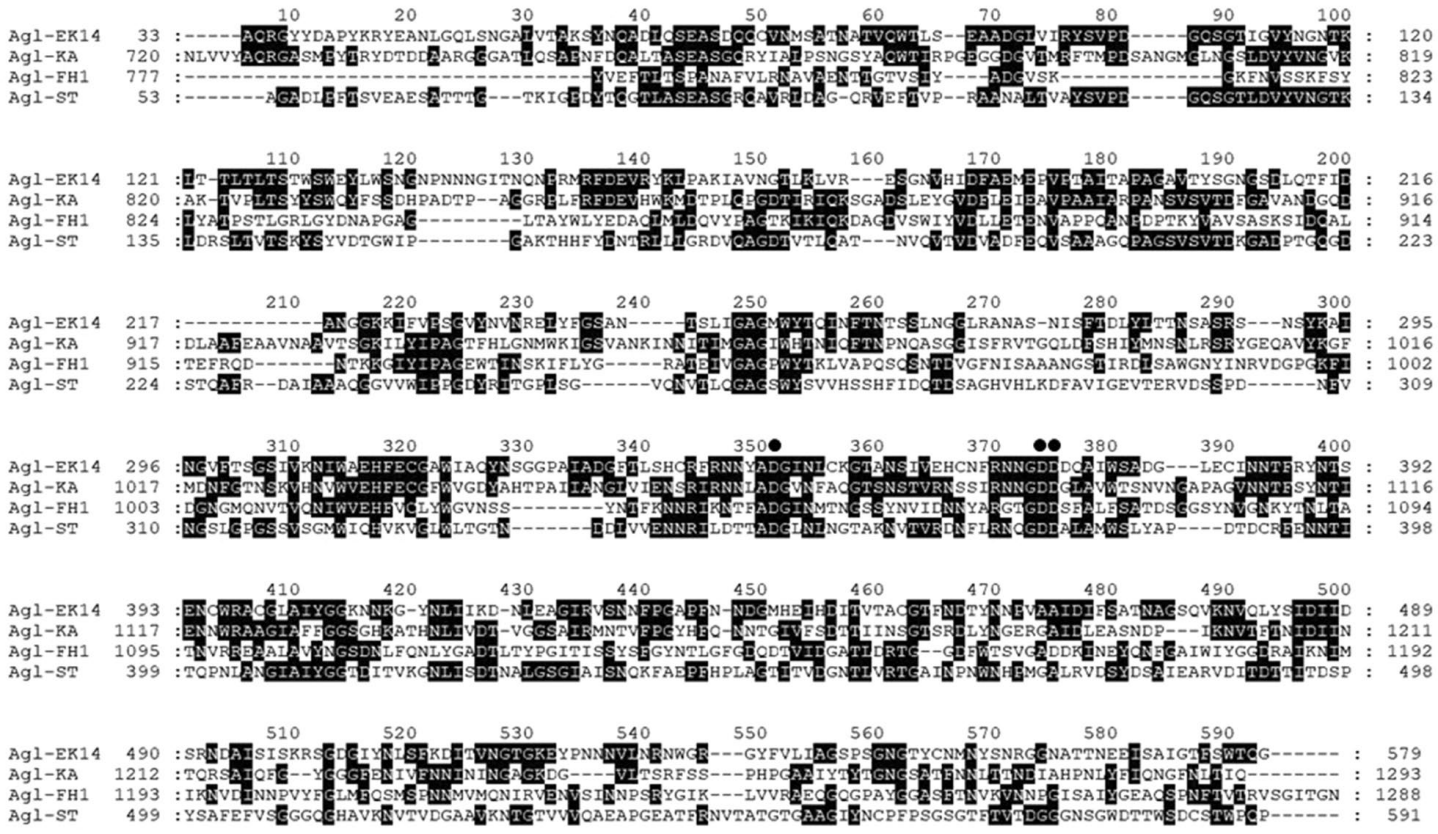
Multiple alignments of amino acid sequences of the catalytic domain of Agl-EK14 with other GH87 α-1,3-glucanases. Amino acid sequences were aligned by ClustalW. The residue numbers of the first and last amino acids in each line are shown on the left and right. The black box indicates the same amino acids. Amino acids conserved in at least three of the four sequences are indicated by a gray box. Amino acids conserved in at least two of the four sequences are indicated by a light gray box. Three Asp residues at the catalytic site of GH87 enzymes are indicated by solid circles.

Multiple alignments of catalytic domains of various GH87 enzymes showed that the domain of *Flavobacterium* sp. EK-14 α-1,3-glucanase displays sequence similarities (30%, 17%, and 23%, respectively) to the domain of Agl-KA from *B. circulans* KA-304, Agl-FH1 of *P. glycanilyticus* FH11, and Agl-ST of *S. thermodiastaticus* HF3-3 (Fig. 2). The catalytic domain of *Flavobacterium* sp. EK-14 α-1,3-glucanase showed three conserved Asp residues participating in the reaction catalyzed by GH87 α-1,3-glucanase^25^. Based on site-directed mutagenesis and crystal structure analysis, it was proposed that D1068 of Agl-FH1 from *P. glycanilyticus* FH11 acted as the general acid catalyst and a proton donor to the glycosidic bond, whereas D1045 or D1069 acted as the general base catalyst and activated the nucleophilic water^25^. The D1045, D1068, and D1069 of Agl-FH1 corresponded to the D346, D369, and D370 of Agl-EK14, respectively.

A phylogenetic tree was constructed based on the amino acid sequences of the catalytic domains of GH87 α-1,3-glucanases and mycodextranases. The phylogenetic tree revealed that Agl-EK14 was distinct from several clusters containing enzymes that have already been cloned and characterized (Supplementary Fig. S4).

### Expression and purification of recombinant Agl-EK14

To clarify enzymatic properties, the Agl-EK14 gene excluding the signal peptide and CTD, was expressed in *E. coli* (Fig. 1A). The expression plasmid pET-aglEK14 was introduced into *E. coli* Rosetta-gami B (DE3) cells. The α-1,3-glucanase activity of the cell-free extract of *E. coli* harboring the plasmid was 29.6 units/mg. The recombinant enzyme was purified from cell-free extracts using anion exchange and hydrophobic interaction column. Figure 1B shows the sodium dodecyl sulfate–polyacrylamide gel electrophoresis (SDS-PAGE) analysis of purified Agl-EK14. The predicted molecular mass of Agl-EK14 was 92,970, and its molecular mass, as estimated by SDS-PAGE, was consistent with the predicted values. The purified Agl-EK14 exhibited a specific activity of 134 units/mg (Supplementary Table S2).

### Properties of Agl-EK14

**Figure 3:**
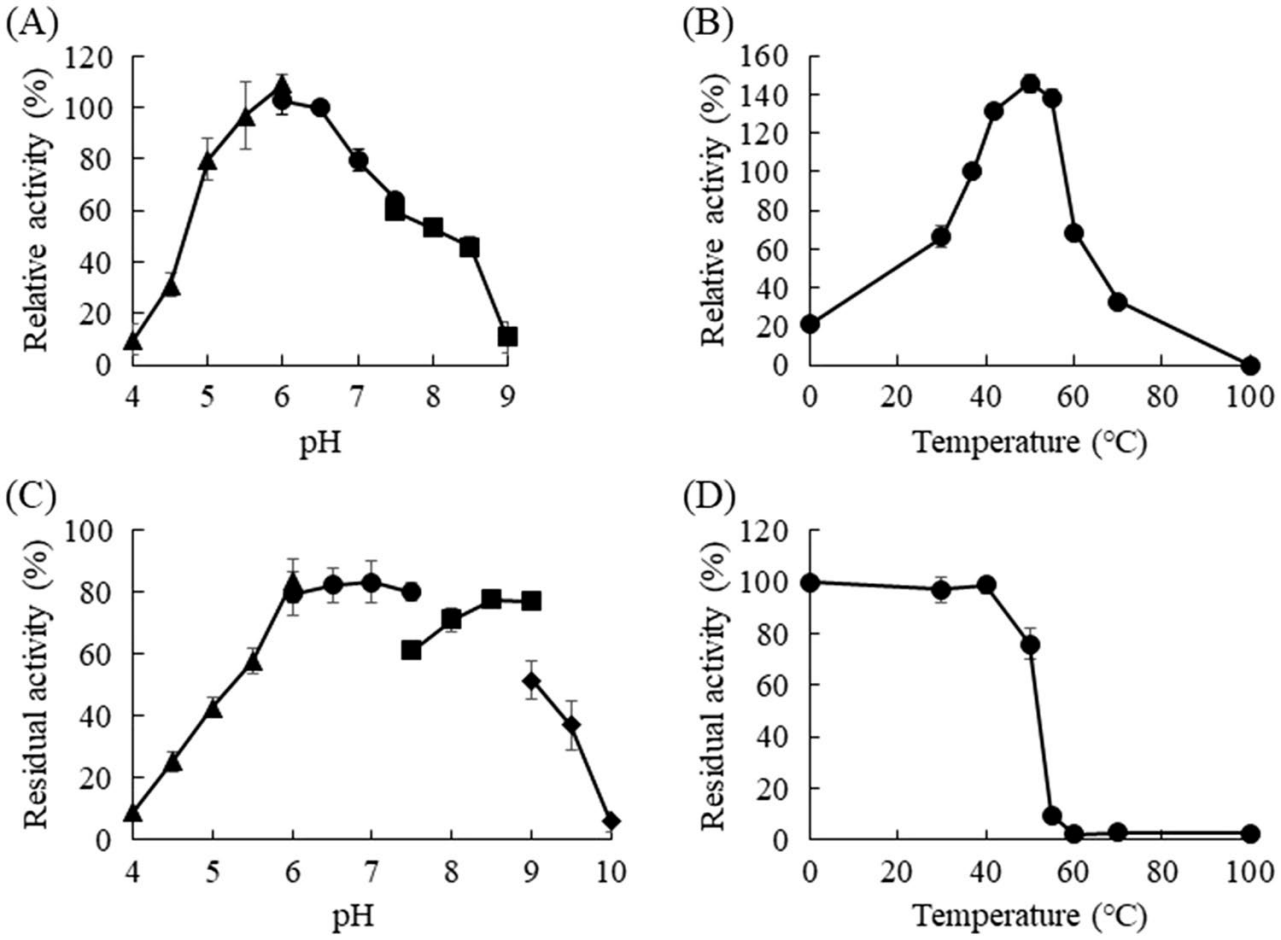
Optimum pH (**A**), optimal temperature (**B**), pH stability (**C**), and thermal stability (**D**) of Agl-EK14. The activity values are the mean ± standard deviation of three independent experiments. Symbols: closed triangles, sodium acetate buffer; closed circles, potassium phosphate buffer; closed squares, Tris–HCl buffer; and closed diamonds, borate buffer.

Agl-EK14 exhibited maximum activity at pH 6.0 in sodium acetate buffer (Fig. 3A) and an optimal temperature at 50 °C (Fig. 3B). Agl-EK14 was stable at pH 6.0–9.0 and maintained more than 60% of maximum activity after incubation at 50 °C for 30 min (Fig. 3C). Agl-EK14 retained full activity after 30 min of incubation at 40 °C and maintained more than 75% of its maximum activity at 50°C (Fig. 3D). The substrate specificity of Agl-EK14 was measured using various polysaccharides (Supplementary Table S3). Agl-EK14 hydrolyzed α-1,3-glucan but scarcely on starch (main linkage-type; α-1,4-), dextran (α-1,6-), laminarin (β-1,3-), and cellulose (β-1,4-).

### α-1,3-Glucan hydrolytic activity

**Figure 4:**
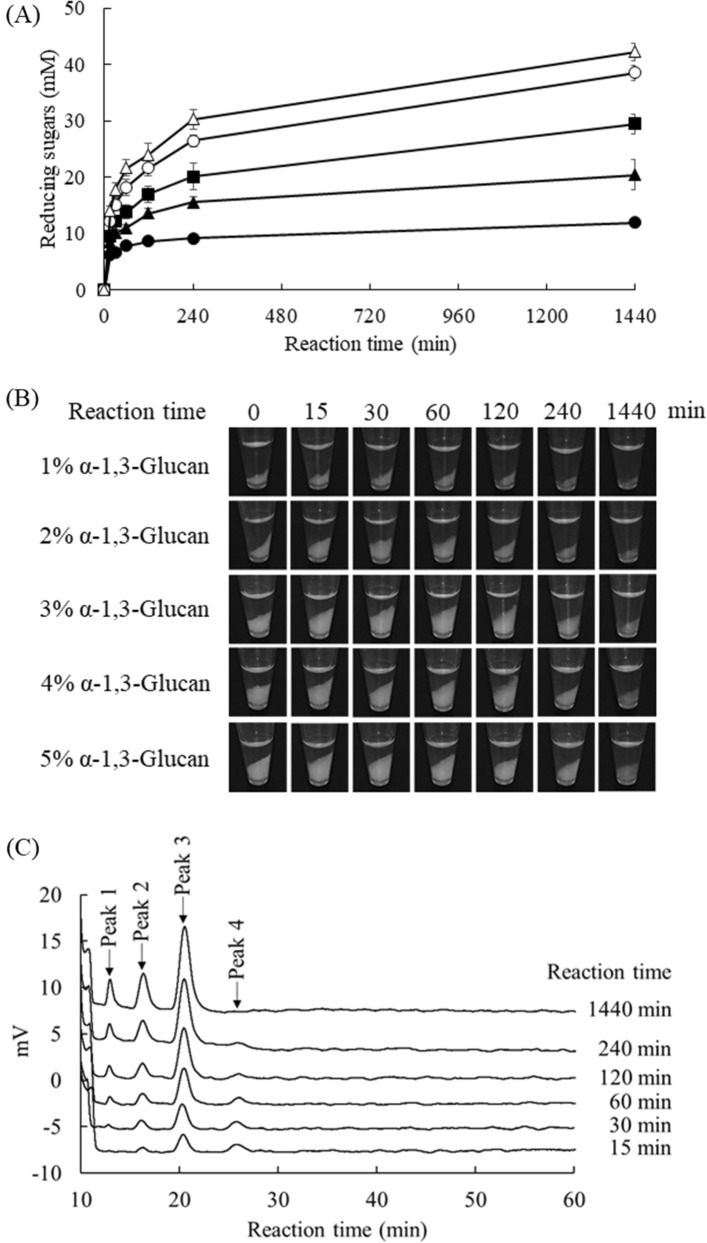
Effect of substrate concentration on α-1,3-glucan hydrolysis of Agl-EK14 (**A**), observation of reaction residues (**B**), and HPLC analysis of the product from 1% α-1,3-glucan (**C**). The reaction mixtures containing 1.0 nmol/ml Agl-EK14, 50 mM potassium phosphate (pH 6.5), and appropriate amounts of α-1,3-glucan were incubated at 37 °C. (**A**) Each data point represents the mean ± standard deviation from triplicate experiments. Symbols: solid circles, 1% α-1,3-glucan; solid triangles, 2% α-1,3-glucan; solid squares, 3% α-1,3-glucan; open circles, 4% α-1,3-glucan; open triangles, 5% α-1,3-glucan. (**B**) The reaction was stopped by boiling for 10 min. After centrifugation at 13,000 rpm for 10 min, photographs of the reaction mixture were taken. (**C**) Hydrolytic products were separated from insoluble α-1,3-glucan by centrifugation and were analyzed by HPLC.

Figure 4A shows the effect of substrate concentration on the glucan hydrolysis of Agl-EK14. The concentration of reducing sugars increased with increasing α-1,3-glucan, and approximately 12.0, 20.4, 29.5, 38.6, and 42.2 mM reducing sugars were released from 1%, 2%, 3%, 4%, and 5% α-1,3-glucan, respectively, after 1440 min of reaction. Agl-KA from *B. circulans* KA-304 is one of the most investigated α-1,3-glucanases, and its various properties have been well characterized. Compared to the hydrolytic activity of Agl-KA on α-1,3-glucan, the amounts of reducing sugars released by Agl-EK14 were almost 1.5-fold higher than those by Agl-KA after 24 h of reaction (Supplementary Fig. S5). After the reaction, the residues were collected by centrifugation (Fig. 4B). The residues of the reaction mixture with 1% and 2% substrates were almost negligible after 1440 min. Reaction products released from 1% α-1,3-glucan were analyzed by high-performance liquid chromatography (HPLC; Fig. 4C), and four peaks were detected. Because the retention time of peak 1 coincided with glucose, electrospray ionization-mass spectrometry (ESI–MS) analysis was performed against peaks 2–4 (Supplementary Fig. S6). Results revealed that peaks 2–4 were assigned to the [M + Na]^+^ ions of di-, tri-, and tetra-saccharide at 365.0, 527.0, and 689.1 *m/z*, respectively. These results suggested that Agl-EK14 released di- to tetra-saccharides after a 15-min reaction. Tetra-saccharides gradually decreased, but glucose, di-, and tri-saccharides increased during incubation.

### Hydrolysis of the fungal cell wall α-1,3-glucan

**Figure 5:**
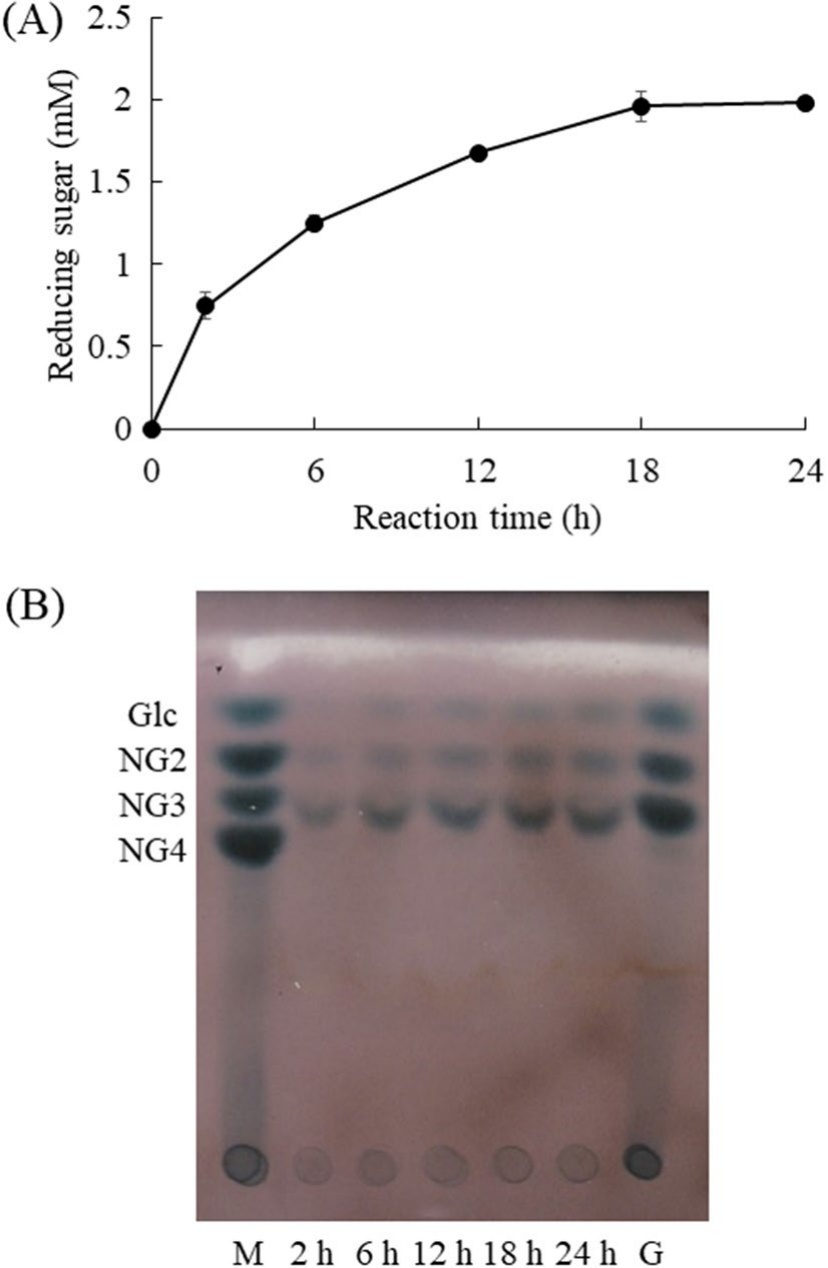
Hydrolysis of the cell wall preparation of *A. oryzae* (**A**) and TLC analysis of reaction products from the cell wall preparation (**B**). The reaction mixture contained 2% cell wall preparation of *A. oryzae*, 50 mM potassium phosphate (pH 6.5), and 1.0 nmol/ml Agl-EK14. The mixture was incubated at 37 °C. (**A**) Each data point represents the mean ± standard deviation from triplicate experiments. (**B**) The 2–24h reaction products were spotted at 2 μl on TLC. TLC was performed as described in Methods. Glucose (Glc), nigerose (NG2), nigerotriose (NG3), and nigerotetraose (NG4) were used as markers. Lane M, markers; lane G, α-1,3-glucan hydrolytic products of Agl-EK14 after 24 h.

The cell wall of *A. oryzae* is composed of cross-linked polysaccharides and glycoproteins. Chitin and β-glucan are the main skeletal polysaccharides, and α-1,3-glucan is mainly localized on the cell wall surface^8^. The cell wall preparation of *A. oryzae* included polysaccharide complexes containing α-1,3-glucan, and the hydrolytic activity of Agl-EK14 was investigated against the cell wall preparation. Figure 5A shows the time course of hydrolysis of the cell wall preparation of Agl-EK14. Agl-EK14 released approximately 2.0 mM reducing sugars after a 24 h hydrolysis reaction. The reaction products were analyzed by thin-layer chromatography (TLC; Fig. 5B), and mono-, di-, and tri-saccharides were detected. These reaction products were consistent with hydrolytic products from insoluble α-1,3-glucan synthesized by GTF-I of *S. mutans*.

**Figure 6:**
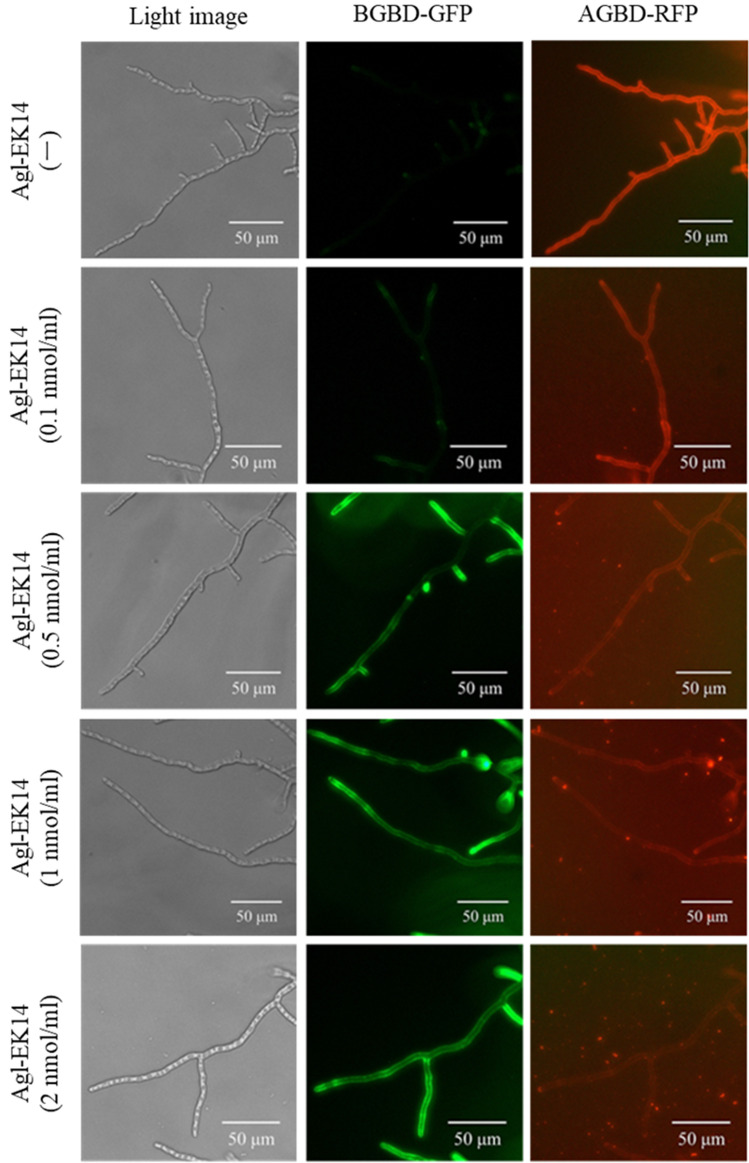
Cell wall binding assay of BGBD-GFP and AGBD-RFP for *A. oryzae* live mycelia.

In a previous study, *A. oryzae* live mycelia were treated with α-1,3-glucanase Agl-KA of *B. circulans* KA-304, and mycelia were stained with the green fluorescent protein (GFP)-fused β-1,3-glucan binding domain of *Lysobacter* sp. MK9-1 (BGBD-GFP)^38^. In contrast, *A. oryzae* live mycelia without enzyme treatment were not stained with BGBD-GFP. To confirm the hydrolytic activity of Agl-EK14 against the cell wall surface α-1,3-glucan, *A. oryzae* live mycelia were treated with Agl-EK14, and mycelia were stained with BGBD-GFP after treatment. As a result, mycelia treated with Agl-EK14 (more than 0.5 nmol/ml) were stained with BGBD-GFP (Fig. 6). To determine the residual α-1,3-glucan on the cell wall surface layer, mycelia treated with Agl-EK14 were also stained with red fluorescent protein (RFP)-fused α-1,3-glucan binding domains of Agl-KA from *B. circulans* KA-304 (AGBD-RFP)^39^. AGBD-RFP strongly bound to mycelia without Agl-EK14 treatment but scarcely bound to mycelia treated with Agl-EK14 (more than 0.5 nmol/ml). Furthermore, the same experiment was performed with Agl-KA from *B. circulans* KA-304. As a result, BGBD-GFP bound to the entire mycelia at 2.0 nmol/ml Agl-KA, and AGBD-RFP did not bind (Supplementary Fig. S7). These results suggested that Agl-EK14 hydrolyzed α-1,3-glucan of the cell wall more efficiently compared to Agl-KA.

**Figure 7:**
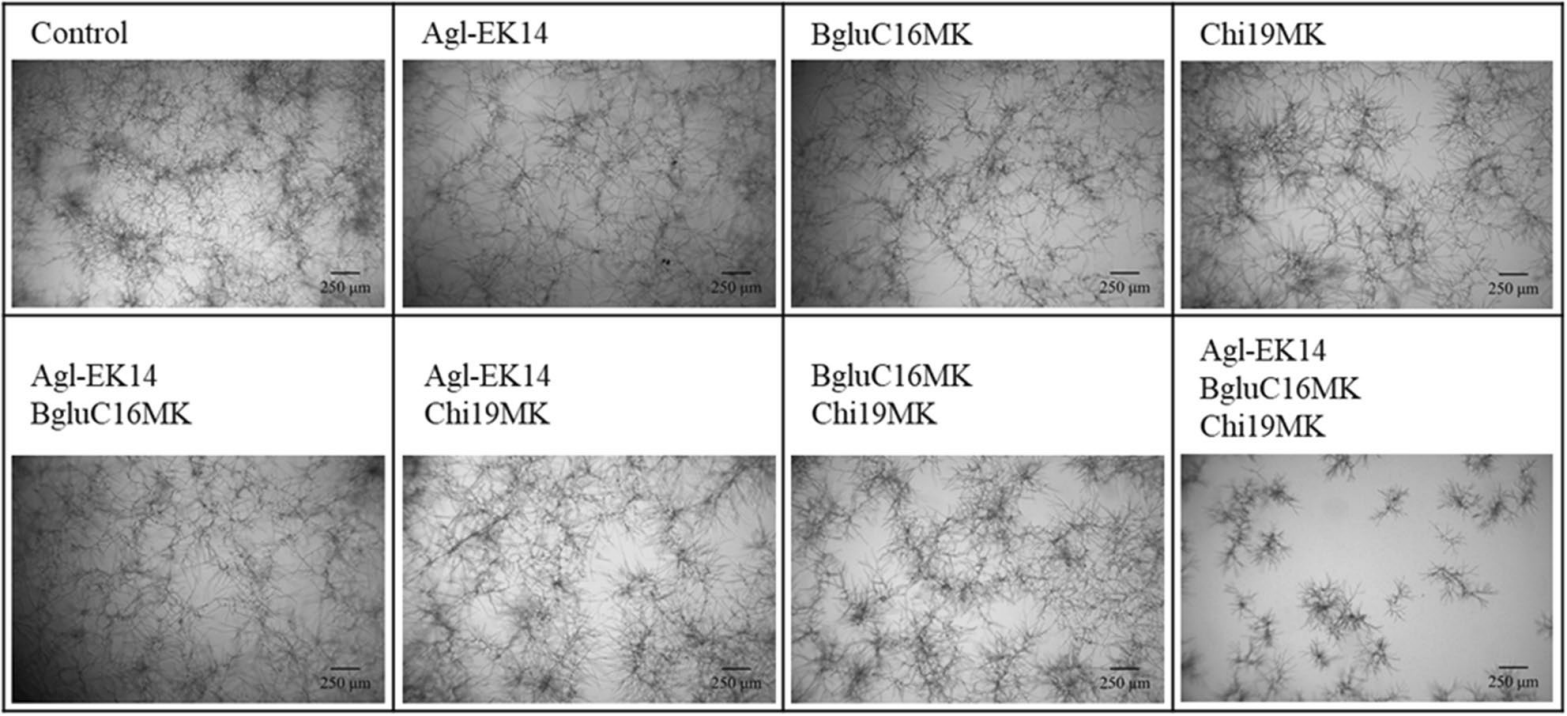
Growth inhibition assay of Agl-EK14 for *A. oryzae* live mycelia.

GH19 chitinase (Chi19MK) from *Lysobacter* sp. MK9-1 inhibited the growth of fungi such as *Trichoderma reesei*, *Trichoderma viride*, and *Shizophyllum commune*. GH16 β-1,3-glucanase (BgluC16MK) of *Lysobacter* sp. MK9-1 enhanced the growth-inhibitory activity of Chi19MK against* T. reesei*^38,40^. This study investigated the growth inhibition assay of Agl-EK14 with Chi19MK and BgluC16MK against *A. oryzae* (Fig. 7). As a result, a mixture of Chi19MK and BgluC16MK did not inhibit the growth of *A. oryzae*, but the addition of Agl-EK14 to the mixture inhibited the mycelial growth of *A. oryzae*. In the case of the mixture containing Agl-EK14, Chi19MK, and BgluC16MK, the colony diameter was 380–820 μm, and the colonies existed individually (Supplementary Fig. S8). In contrast, under other conditions, colonies were extended and connected, and individual colony diameters could not be measured.

## Discussion

The genus *Flavobacterium* comprises extracellular polysaccharide-assimilating bacteria and produces extracellular polysaccharide-degrading enzymes. For example, *F. johnsoniae* can digest extracellular insoluble chitin by secreting some chitinases^41^. As for extracellular secreted β-glucan-degrading enzymes, yeast cell wall lytic β-1,3-glucanases from *Flavobacterium dormitator*^42^ and a low-temperature active β-1,3-glucanase from *Flavobacterium* sp. strain 4221 have been reported^43^. Among these studies, *Flavobacterium* sp. EK-14 was isolated as a producer of α-1,3-glucanase.

In this study, the gene encoding GH87 α-1,3-glucanase (Agl-EK14) was cloned from *Flavobacterium* sp. EK-14 based on putative α-1,3-glucanase genes in the database. Agl-EK14 strongly hydrolyzed α-1,3-glucan, and the reaction residues from 1 to 2% substrates almost disappeared after 1 day of reaction (Fig. 4A,B). At this point, the turbidity of the reaction mixtures clouded by insoluble α-1,3-glucan decreased and gradually became clear. Although α-1,3-glucanase Agl-KA from *B. circulans* KA-304 was studied over a prolonged period, hydrolysis of the most insoluble glucan was never observed. Agl-EK14 was stable over a wide pH range and inactive on substrates except for α-1,3-glucan. These properties were similar to those of α-1,3-glucanases investigated for removing dental plaque, such as MutP from *Paenibacillus curdlanolyticus* MP-1^44^, Agl-ST1 and Agl-ST2 from *S. thermodiastaticus* HF3-3^45^, and mutanase RM1 from *Paenibacillus* sp. RM1^46^. Although further studies on plaque inhibition assay and stability against toothpaste additives are required, these results suggest that Agl-EK14 has potential as a plaque removal enzyme.

Furthermore, Agl-EK14 hydrolyzed the cell surface α-1,3-glucan of *A. oryzae* (Fig. 6). After treatment of the live mycelia of *A. oryzae* with Agl-EK14, BGBD-GFP bound to the cell wall. In some of the mycelial tips of *A. oryzae*, BGBD-GFP bound firmly. At the mycelial tip, the synthesis and degradation of cell wall polysaccharides proceed simultaneously^47^. The mycelial tip was probably in the process of becoming a complex structure, such as a multi-layered structure of glycans and cross-linked structures between glycans. Thus, BGBD-GFP could readily bind. Although AGBD-RFP bound strongly to the cell surface before Agl-EK14 treatment, AGBD-RFP hardly bound after the treatment. Because α-1,3-glucan was located on the cell surface and not the main skeletal polysaccharide, only α-1,3-glucanase treatment hardly influenced the growth and fungi form. The combination of Agl-EK14 with chitinase Chi19MK and β-glucanase BgluC16MK inhibited the growth of *A. oryzae* (Fig. 7), suggesting that their combination provides a potent antifungal enzyme preparation.

As mentioned in the Introduction, α-1,3-glucanase, an EK-14 enzyme, was purified from a culture filtrate of *Flavobacterium* sp. EK-14, and some properties of the EK-14 enzyme were reported^27^. Some enzymatic properties of the EK-14 enzyme, such as optimum pH, optimal temperature, and substrate specificity, were almost similar to those of Agl-EK14 obtained in this study. However, there were some inconsistencies between the EK-14 enzyme and Agl-EK14. The molecular weight of the EK-14 enzyme was about 65,000, and the specific activity of the EK-14 enzyme was 0.813 units/mg. In contrast, the molecular weight of Agl-EK14 was 92,970, and the specific activity was 134 units/mg, implying that Agl-EK14 is an isozyme of the EK-14 enzyme.

Although the relation between the previously purified EK-14 enzyme and cloned Agl-EK14 based on the DNA sequence is yet to be clarified, this study revealed that Agl-EK14 has excellent α-1,3-glucan-degrading activity and forms a cluster independent of known α-1,3-glucanases from gram-positive bacteria. Hence, Agl-EK14 is a valuable enzyme to be studied for industrial applications. Functional and structural analyses are in progress for further improvement of this enzyme.

## Methods

### Materials

α-1,3-Glucan was prepared as described previously^48^. Nigerose, nigerotriose, and nigerotetraose were prepared by enzyme hydrolysis of α-1,3-glucan as described previously^49^. Starch (potato), dextran, and laminarin were purchased from Nacalai Tesque, Inc. (Kyoto, Japan). Microcrystalline cellulose was purchased from Sigma-Aldrich Japan (Tokyo, Japan). The other reagents were chemically pure-grade commercial products.

### Microorganisms and culture

*Flavobacterium* sp. EK-14 was grown at 22 °C in trypticase soy broth. *A. oryzae* NBRC 100959 was cultured at 28 °C in a potato dextrose agar (PDA) medium. The *A. oryzae* conidial solution was prepared by suspending conidia formed in the PDA medium in a suspension buffer containing 0.01% Tween 20 and 100 mM NaCl. The conidial solution was inoculated into a Czapek-Dox (CD) medium for the cell wall binding assay and incubated at 28 °C.

### Cell wall preparation of* A. oryzae*

*A. oryzae* live mycelia (from a 5-day culture with 2 L CD medium) were suspended in 100 ml acetone. Mycelia were collected by filtration and air-dried. Acetone-dried mycelia were suspended in water and heated at 120 °C for 20 min in an autoclave. Autoclaved mycelia were washed with distilled water and lyophilized. Lyophilized mycelia were used as cell wall preparation of *A. oryzae*.

### Cloning of the α-1,3-glucanase gene

All primers used in cloning the Agl-EK14 gene are listed in Supplementary Table S1. PCR amplified the partial Agl-EK14 gene from the genomic DNA of *Flavobacterium* sp. EK-14. The sense primer, Flavo-for, was designed from a conserved region of putative GH 87 α-1,3-glucanase genes from *F. anhuiense* M168 (accession no. CP023642.1; region: 32,333,180–2,336,068) and *F. johnsoniae* UW101 (accession no. CP000685.1; region: 3,818,600–3,821,545). The antisense primer, Flavo-rev, was designed from their conserved regions. The DNA fragment containing upstream and downstream regions of the partial Agl-EK14 gene was amplified by PCR using the primer FlavoEK-f4 and FlavoEK-r4. The FlavoEK-f4 was designed based on the upstream region (position at − 190 to − 156) of the ORF of the *F. anhuiense* strain M168 α-1,3-glucanase gene. The FlavoEK-r4 was designed based on the downstream region (position at 3081 to 3115) of the ORF of the *F. anhuiense* gene. The nucleotide sequence of the Agl-EK14 gene from *Flavobacterium* sp. EK-14 was assigned to the DNA Data Bank of Japan (DDBJ; https://www.ddbj.nig.ac.jp/index.html), Accession No. LC735653.

### Expression of the Agl-EK14 gene

The ORF, excluding the signal peptide, was amplified by PCR from the genomic DNA of *Flavobacterium* sp. EK-14 using primers the Flavof1RE and Flavo5rRE. The PCR fragment was digested with Nde I and BamH I; the resulting fragment was cloned into pET22b (+); and the vector was designated as pET-aglEK14CTD. Subsequently, the gene, excluding CTD, was amplified from pET-aglEK14CTD using primers the T7 promoter and Cbam4. The PCR fragment was digested with Nde I and BamH I, and the resulting fragment was cloned into pET22b (+). The plasmid expressing Agl-EK14 was designated as pET-aglEK14.

The constructed plasmid was transformed into *E. coli* Rosetta-gami B (DE3) cells, and the resulting transformant was inoculated into a 2 l flask containing 1 L LB medium with ampicillin, chloramphenicol, and kanamycin incubated at 30 °C on a reciprocal shaker (100 strokes/min). After incubation, until the optical density at 600 nm was about 0.6, isopropyl-β-d-thiogalactopyranoside was added to the culture medium at a final concentration of 0.4 mM. The culture was incubated at 16 °C for an additional 18 h.

### Purification of Agl-EK14

*E. coli* Rosetta-gami B (DE3) cells harboring pET-aglEK14 were harvested from 1 l culture medium by centrifugation (5000×*g* for 10 min). Cells were suspended in 10 mM potassium phosphate buffer (pH 7.0) and disrupted by sonication (4 °C, 10 min, 20 kHz, 20–40 W) on ice. Each cell’s debris was removed by centrifugation (8000×*g* for 10 min). The supernatant was dialyzed against the buffer used for cell suspension.

To purify Agl-EK14, the dialysate (cell-free extract) was fractionated with ammonium sulfate, and the fraction of 25–70% saturation was dialyzed against 10 mM potassium phosphate (pH 7.0). The dialysate was applied to a Cellufine A-500 column (2.0 × 4.5 cm) equilibrated with 10 mM potassium phosphate buffer (pH 7.0). After washing with the same buffer, Agl-EK14 was found in the unabsorbed fractions. The activity fraction was mixed with ammonium sulfate at 25% saturation and applied to a HiTrap Butyl 650M column (1.5 × 2.5 cm) equilibrated with 10 mM potassium phosphate buffer (pH 7.0) containing 25% ammonium sulfate. The column was washed with the buffer containing 25% ammonium sulfate, and Agl-EK14 was eluted by linearly decreasing the ammonium sulfate concentration from 25 to 0%. The fraction containing Agl-EK14 was dialyzed against 10 mM potassium phosphate buffer (pH 7.0).

### Assay of α-1,3-glucanase activity

α-1,3-Glucanase activity was measured as described previously^48^. The reaction mixture contained 50 mM potassium phosphate buffer (pH 6.5), 1% α-1,3-glucan, and appropriate amounts of enzyme. The reaction mixture was incubated at 37 °C. One unit of enzyme is defined as the amount of enzyme that releases 1 μmol of reducing sugar per minute.

### Effect of temperature on the activity and stability of α-1,3-glucanase

The optimal temperature for Agl-EK14 (0.005 nmol/ml) activity was determined by monitoring the amount of reducing sugar produced between 0 and 100 °C for 30 min in 50 mM potassium phosphate buffer (pH 6.5). To determine thermal stability, Agl-EK14 (0.1 nmol/ml) in 50 mM potassium phosphate buffer (pH 6.5) was treated at various temperatures ranging from 0 to 100 °C for 30 min. After temperature treatment, α-1,3-glucanase activity was measured using 0.005 nmol/ml of the treated enzyme in 50 mM potassium phosphate buffer (pH 6.5) at 37 °C.

### Effect of pH on the activity and stability of α-1,3-glucanase

The optimal pH for α-1,3-glucanase activity was determined by incubating a mixture containing 1% α-1,3-glucan, 0.005 nmol/ml α-1,3-glucanase, and 50 mM buffer of different pH values at 37 °C for 10 min. The buffers used were sodium acetate (pH 4.0–6.0), potassium phosphate (pH 6.0–7.5), Tris–HCl (pH 7.5–9.0), and sodium borate (pH 9.0–10.0). The same buffers were used to analyze pH stability by incubating the enzyme (0.1 nmol/ml) in 50 mM buffer at 50 °C for 30 min. After treatment, α-1,3-glucanase activity was measured using 0.005 nmol/ml of the treated enzyme in 50 mM potassium phosphate buffer (pH 6.5) at 37 °C.

### Hydrolysis of α-1,3-glucan in the cell wall of *A. oryzae* live mycelia

The conidial solution of *A. oryzae* at a final concentration of 10^3^ cells/ml was added to the CD medium in a 24-well plate. Agl-EK14 at 0–2 nmol/ml was added to the culture. Cells were incubated at 30 °C for up to 30 h. After incubation, mycelia were washed with 10 mM Tris–HCl (pH 8.0), and the washed mycelia were treated with α-1,3-glucan binding domain fused with RFP (AGBD-RFP; referred to as DCD-tetraRFP in the previous study)^39^ and β-1,3-glucan binding domain fused with GFP (BGBD-GFP)^38^. AGBD-RFP strongly bound to α-1,3-glucan and stained α-1,3-glucan in the cell wall of *A. oryzae* live mycelia. BGBD-GFP stained β-1,3-glucan in the cell wall of *A. oryzae* live mycelia. Mycelia treated with AGBD-RFP (1.0 nmol/ml) and BGBD-GFP (1.0 nmol/ml) were observed using an Olympus CKX53 fluorescence microscope (Tokyo, Japan).

The growth inhibition assay against *A. oryzae* was performed with GH 19 chitinase (Chi19MK) and GH 16 β-1,3-glucanase (BgluC16MK) of *Lysobacter* sp. MK9-1^38,40^. Washed mycelia were treated with Chi19MK (3.0 nmol/ml), BgluC16MK (3.0 nmol/ml), and Agl-EK14 (3.0 nmol/ml). After enzyme treatment, mycelia were observed under a microscope.

### Analytical methods

Protein concentrations were measured during enzyme purification using Lowry’s method^50^, with bovine serum albumin as the standard. Otherwise, the protein concentration of purified Agl-EK14 was estimated by measuring the absorbance at 280 nm with a molar absorption coefficient of 165,880 M^–1^ cm^–1^^51^. AGBD-RFP concentration was estimated by measuring the absorbance at 554 nm with a molar absorption coefficient of 35,600 M^−1^ cm^−1^. The BGBD-GFP concentration was estimated by measuring the absorbance at 475 nm with a molar absorption coefficient of 32,500 M^−1^ cm^−1^. SDS-PAGE was performed using Laemmli’s method^52^. PM1500 Excel Band All Blue Regular Range Plus Protein Marker (purchased from SMOBIO, Taiwan) was used as a molecular marker. Reducing sugars were quantified with dinitrosalicylic acid reagent using the Miller method, and glucose (0–4 mM) was used as the standard^53^. HPLC was used to analyze the products of hydrolysis. The reaction products were applied onto a column of Inertsil amide (4.6 × 250 mm; GL Sciences, Inc., Tokyo, Japan), and the products were monitored using an RID-10A refractive index detector (Shimadzu Co., Kyoto, Japan). The elution solvent was 70% acetonitrile at a flow rate of 0.5 ml/min with LC-10AT pumps (Shimadzu). The hydrolytic products from the cell wall preparation of *A. oryzae* were analyzed by TLC as described previously^49^.

### Supplementary Information

Supplementary Information.

## Data Availability

All the data are available in the article and the online supplementary material. Further inquiries can be directed to the corresponding author.
