# Supplementary material for: α-1,3-Glucanase from the gram-negative bacterium Flavobacterium sp. EK-14 hydrolyzes fungal cell wall α-1,3-glucan
Source: Sci Rep. 2023 Dec 5;13:21420. doi: 10.1038/s41598-023-48627-y (PMC10696023; doi:10.1038/s41598-023-48627-y)
Supplement: Supplementary file 1 — Supplementary Information. [file 41598_2023_48627_MOESM1_ESM.pdf]

Supplementary information

Title: α-1,3-Glucanase from the gram-negative bacterium *Flavobacterium* sp. EK-14 hydrolyzes fungal cell wall α-1,3-glucan

Masaki Takahashi[1], Shigekazu Yano[1*], Yui Horaguchi[1], Yuitsu Otsuka[1], Wasana Suyotha[2], Koki Makabe[1], Hiroyuki Konno[1], and Susumu Kokeguchi[3]

[1]*Graduate School of Sciences and Engineering, Yamagata University* (*Jonan, Yonezawa, Yamagata 992-8510, Japan*)

[2]*Enzyme Technology Laboratory, Faculty of Agro-industry, Prince of Songkla University (Hat Yai 90112, Thailand*)

[3]*Department of Oral Microbiology, Graduate School of Medicine, Dentistry and Pharmaceutical Sciences, Okayama University (Okayama 700-0914, Japan)*

Supplementary Table S1. Oligonucleotide primers.

Primer name	Oligonucleotide sequence
Flavo-for	5′-TATGCSCAGCGMGGTTATTATGATGCASCTTATAA-3′
Flavo-rev	5′-TTTACYAAAACTTTTCTTCCGYTKMTATCWGTAAC-3′
FlavoEK-f4	5′-TTGCATCTATATTGATTCTTGATTTTTTATTTTGG-3′
FlavoEK-r4	5′-GCACGGATTACAAATCCGCGCTATCGTTGTTGAGC-3′
Flavof1RE	5′-TGCTTCAGGTTCCCATATGCAGCGCGGTTATTATG-3′
Flavo5rRE	5′-CTAAATTTTGGATCCTCATTTAATAATCAATTTTT-3′
T7 promoter	5′-CCCGCGAAATTAATACGACTCACTATAGGG-3′
Cbam4	5′-GGAAGGATTGGATCCTTAGCTAACTACAGTATTCT-3′

Restriction sites in the oligonucleotide sequences are underlined.

Supplementary Table S2. Purification of Agl-EK14

	Volume (ml)	Total activity (units)	Total protein (mg)	Specific activity (units/mg)	Yield (%)
Cell-free extract	50	9500	321	29.6	100
$(NH_4)_2SO_4$ precipitation 25–70% fraction	30	6270	171	36.8	66.1
Cellufine A-500	93	5950	63.9	93.1	62.6
HiTrap Butyl 650M	31	4310	32.1	134	45.4

Supplementary Table S3. Substrate specificity of Agl-EK14

Substrate	Main linkage-type	Relative activity (%)
α-1,3-Glucan	α-1,3-	100
Starch	α-1,4-	1.5 ± 0.8
Dextran	α-1,6-	2.0 ± 0.5
Laminarin	β-1,3-	5.0 ± 2.7
Cellulose	β-1,4-	1.3 ± 0.2

Signal peptide (SP)

GH87
Catalytic domain (CAT)

ATGAGAAACAAATTACATTTTATTTTTTTGAGAAAAGCCCAATATTACCATGTTATATTA 60
M R N K L H F I F L R K A Q Y Y H V I L

TGGCTTTTTTTTCTTCTTTTTACTTCAGGTTCCTATGCCCAGCGCGGTTATTATGATGCA 120
W L F F L L F T S G S Y A Q R G Y Y D A

CCTTATAAAAGGTACGAAGCAAATTTAGGTCAATTGTCTAATGGTGCTTTAGTTACTGCA 180
P Y K R Y E A N L G Q L S N G A L V T A

AAATCATACAATCAGGCCGATCTTCAGTCAGAAGCTTCAGATCAGCAGTGTGTGAATATG 240
K S Y N Q A D L Q S E A S D Q Q C V N M

TCTGCCACAAACGCGACCGTTCAATGGACGCTAAGTGAAGCTGCCGATGGATTAGTAATT 300
S A T N A T V Q W T L S E A A D G L V I

CGTTATAGTGTTCCTGACGGGCAATCTGGAACTATCGGGGTTTATAATGGCAACACAAAG 360
R Y S V P D G Q S G T I G V Y N G N T K

CTTACAACGCTTACTTTGACTTCAACTTGGTCTTGGGAATATTTGTGGAGCAACGGAAAT 420
L T T L T L T S T W S W E Y L W S N G N

CCGAACAATAACGGAATTACGAATCAAAACCCAAGAATGCGTTTTGATGAAGTTCGTTAT 480
P N N N G I T N Q N P R M R F D E V R Y

AAACTTCCTGCTAAAATTGCTGTAAACGGAACATTAAAATTGGTGAGAGAATCTGGTAAT 540
K L P A K I A V N G T L K L V R E S G N

GTGCATATTGACTTTGCAGAAATGGAACCAGTGCCAACAGCTATTACAGCTCCTGCAGGT 600
V H I D F A E M E P V P T A I T A P A G

GCCGTAACTTATTCAGGTAACGGAAGCGATCTTCAGACTTTTATTGATGCAAATGGCGGA 660
A V T Y S G N G S D L Q T F I D A N G G

AAAAAAATATTTGTTCCGAGCGGGGTTTATAATGTTAACCGAGAATTATATTTTGGTTCA 720
K K I F V P S G V Y N V N R E L Y F G S

GCAAACACTTCGCTAATTGGTGCAGGAATGTGGTACACACAGATTAATTTTACCAATACT 780
A N T S L I G A G M W Y T Q I N F T N T

AGTAGTTTAAATGGCGGATTACGTGCCAATGCAAGTAACATTTCGTTCACAGATTTATAT 840
S S L N G G L R A N A S N I S F T D L Y

CTCACAACAAATTCAGCTTCGAGAAGTAATTCTTATAAAGCAATAAATGGAGTTTTTACA 900
L T T N S A S R S N S Y K A I N G V F T

AGTGGTTCTATAGTAAAAAATATCTGGGCAGAACATTTTGAATGTGGTGCCTGGATTGCG 960
S G S I V K N I W A E H F E C G A W I A

CAATACAATTCTGGTGGGCCGGCAATTGCTGACGGATTTACATTATCACACTGTCGTTTT 1020
Q Y N S G G P A I A D G F T L S H C R F

AGAAATAATTATGCCGATGGAATCAATCTTTGCAAAGGAACAGCAAATTCAATTGTAGAA 1080
R N N Y A D G I N L C K G T A N S I V E

CACTGTAATTTTAGAAACAACGGAGACGACGATCAGGCGATTTGGTCTGCTGACGGACTC 1140
H C N F R N N G D D D Q A I W S A D G L

GAATGTATCAATAATACATTTCGATACAATACTTCAGAAAACTGTTGGCGCGCTTGTGGA 1200
E C I N N T F R Y N T S E N C W R A C G

CTTGCTATTTATGGAGGGAAAAACAACAAAGGCTACAATTTAATTATCAAAGACAATCTG 1260
L A I Y G G K N N K G Y N L I I K D N L

GAAGCAGGAATTAGAGTAAGTAATAATTTTCCGGGAGCACCGTTTAACAACGATGGTATG 1320
E A G I R V S N N F P G A P F N N D G M

CACGAAATTCATGATATTACCGTAACAGCCTGTGGAACTTTTAATGATACCTACAATAAT 1380
H E I H D I T V T A C G T F N D T Y N N

CCCGTGGCGGCAATTGATATTTTTAGTGCTACAAATGCCGGAAGTCAGGTTAAGAATGTT 1440
P V A A I D I F S A T N A G S Q V K N V

CAGCTTTATAGTATTGATATTATAGATTCAAGAAACGATGCTATTTCTATTAGTAAAAGA 1500
Q L Y S I D I I D S R N D A I S I S K R

TCGGGAGATGGCATTTATAATCTTTCTTTTAAAGACATAACCGTTAACGGAACGGGTAAA 1560
S G D G I Y N L S F K D I T V N G T G K

GAATACCCAAATAACAATGTATTAAACAGAAACTGGGGAAGAGGCTATTTTGTACTTATT 1620
E Y P N N N V L N R N W G R G Y F V L I

```
GCAGGTTCTCCAAGCGGAAATGGTACTTATTGCAATATGAATTATTCTAATAGAGGCGGA 1680
 A  G  S  P  S  G  N  G  T  Y  C  N  M  N  Y  S  N  R  G  G
AATGCCACAACTAATGAAGAAATTAGTGCAATTGGTACATTTTCATGGACGCAGGGCGGA 1720
 N  A  T  T  N  E  E  I  S  A  I  G  T  F  S  W  T  Q  G  G
AACTGTTCCACTACAAATGTCCCGGTTTCTGGTGTAACACTTTCGCCAGGAGCAGTAACT 1780
 N  C  S  T  T  N  V  P  V  S  G  V  T  L  S  P  G  A  V  T
CTAGGTGTTGGTGCTACACAGCAATTAACTCCTGCTGTTTCTCCTGCCAATGCTACCAAT 1860
 L  G  V  G  A  T  Q  Q  L  T  P  A  V  S  P  A  N  A  T  N
AAAACAGTAAGTTATAGTTCAAATAATACTGGTGTTGCTACAGTAAGTGGTTCAGGTTTA 1920
 K  T  V  S  Y  S  S  N  N  T  G  V  A  T  V  S  G  S  G  L
GTAACTGCAGTTGCTTCGGGATCTGCTACAATTACCGTAACGACTCAGGATGGTAATAAA 1980
 V  T  A  V  A  S  G  S  A  T  I  T  V  T  T  Q  D  G  N  K
ACGGCGACTTCTGTAATAACAGTAAATTCATCAAATGTAGCAGTAACAAGTGTGAGTTTA 2040
 T  A  T  S  V  I  T  V  N  S  S  N  V  A  V  T  S  V  S  L
AGTCCTTCATCAGCTACATTAGCGGTAGGCGGAACACAGCAGCTAACACCAACAGTTTTG 2100
 S  P  S  S  A  T  L  A  V  G  G  T  Q  Q  L  T  P  T  V  L
CCTTCAAACGCAACTAATAAATCGGTTAATTATGCTTCAAGCAATACAGGTGTGGCAACG 2160
 P  S  N  A  T  N  K  S  V  N  Y  A  S  S  N  T  G  V  A  T
GTTAATTCATCAGGATTAGTCACTGCAGTTTCAGCAGGTACCGCTACCATTACGGTAACA 2220
 V  N  S  S  G  L  V  T  A  V  S  A  G  T  A  T  I  T  V  T
ACAGTTGATGGAAATAAAACGAGTACTGCAGCAATTACTGTAAATGCCGCAACAGGAAGC 2280
 T  V  D  G  N  K  T  S  T  A  A  I  T  V  N  A  A  T  G  S
TATTTTACAATTAAAAACAAATGGACAGGTAATTATTTATATGATGCCGGAAACAATGTA 2340
 Y  F  T  I  K  N  K  W  T  G  N  Y  L  Y  D  A  G  N  N  V
GGATACGGTCCAACAGTTGCAGACAATACTTATAAATGGGAAAAAGTGGCCATTGATGGT 2400
 G  Y  G  P  T  V  A  D  N  T  Y  K  W  E  K  V  A  I  D  G
ACTTACTATATGATTAAAAATGTAGGTACAGGAGATGTAATGCATATTGAGAATTTAACC 2460
 T  Y  Y  M  I  K  N  V  G  T  G  D  V  M  H  I  E  N  L  T
GGTGCAGTACAATGTACAGCAGGTCAGTCTAGCTGGTGGAGCGCACAATGGTCTTCCGAA 2520
 G  A  V  Q  C  T  A  G  Q  S  S  W  W  S  A  Q  W  S  S  E
AATGTAGACGCAACCTGGGTAAGAATCAAAAACAGATGGCAGACAACAGCAATGATTCAT 2580
 N  V  D  A  T  W  V  R  I  K  N  R  W  Q  T  T  A  M  I  H
ATTGAAAATCTAAATGGTTCTGCACAATATCTTGGTGGTCAAAATAGTTGGGAAAGTGCA 2640
 I  E  N  L  N  G  S  A  Q  Y  L  G  G  Q  N  S  W  E  S  A
CAATGGCAGTTTCAAAACACTTCGACATCGAAAAAAGTGAATCCTGTTGAAGTTGCGGTT 2700
 Q  W  Q  F  Q  N  T  S  T  S  K  K  V  N  P  V  E  V  A  V
GAGAATACTGTAGTTAGCATTTATCCAAATCCTTCCATTGATAATGAATTTAATATTGTT 2760
 E  N  T  V  V  S  I  Y  P  N  P  S  I  D  N  E  F  N  I  V
TTGCCACCACTGGAAGCAGGTGATACAGCAACTGTAACAGTTACTGATAGCAGCGGAAGA 2820
 L  P  P  L  E  A  G  D  T  A  T  V  T  V  T  D  S  S  G  R
AAAGTTTTGGTAAACAAAATTAGTTCATCTTCAAAAATCAGTCATCATTTAGCCCCTGGA 2880
 K  V  L  V  N  K  I  S  S  S  S  K  I  S  H  H  L  A  P  G
ATATATATTGTGACAATTAACTCTAATACATTTAATGTTTCTAAAAAATTGATTATTAAA 2940
 I  Y  I  V  T  I  N  S  N  T  F  N  V  S  K  K  L  I  I  K
TGA                                                          2943
 *
```

First immunoglobulin-like domain (Ig1)

Second immunoglobulin-like domain (Ig2)

Ricin B-like lectin domain (Ricin B)

Carboxy-terminal domain (CTD)

Supplementary Fig. S1. Nucleotide and amino acid sequences of GH87 α-1,3-glucanase (Agl-EK14). The amino acid sequence of the GH87 catalytic domain is shown in light gray; Ig1 and Ig2 are shown in gray; Ricin B is shown in a white box; and CTD is shown in black.

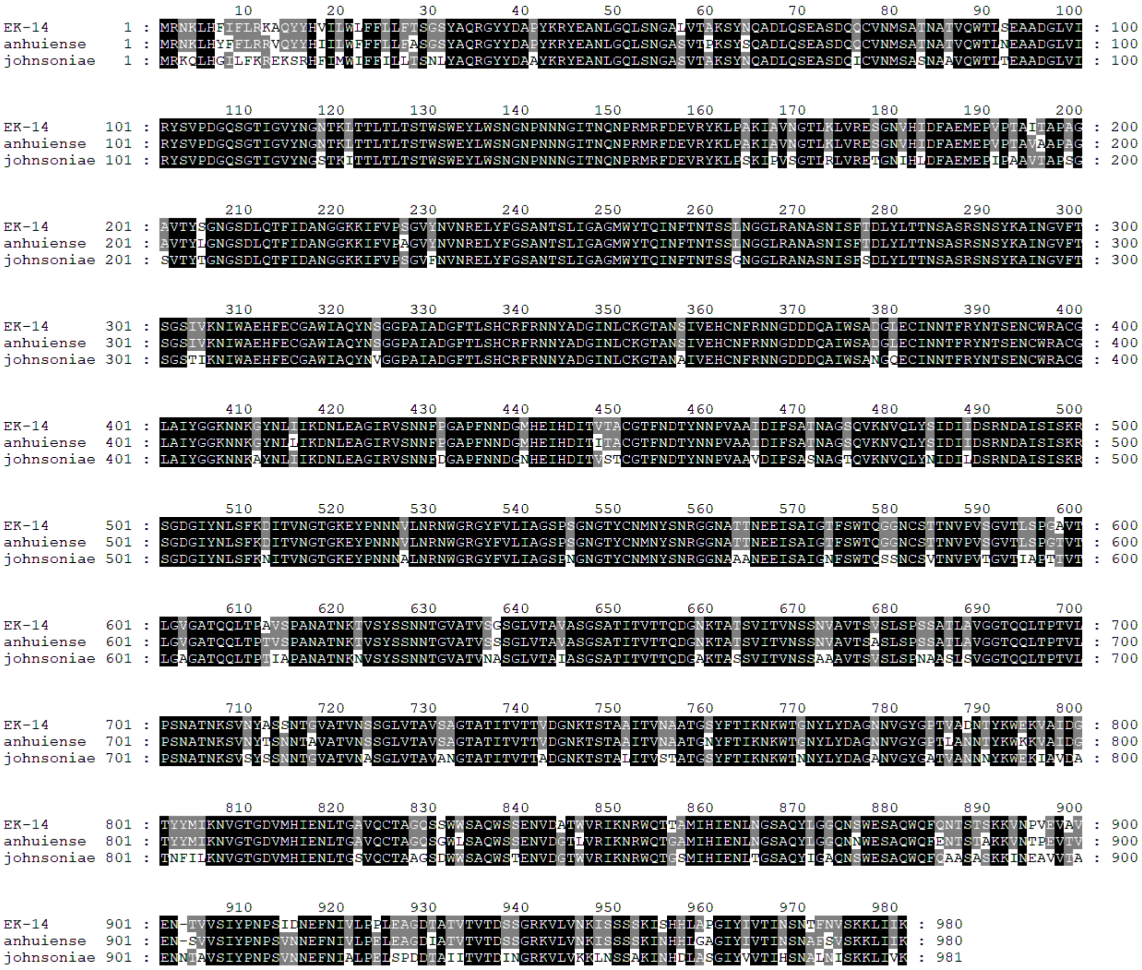

Supplementary Fig. S2. Multiple alignments of amino acid sequences of Agl-EK14 with the putative α-1,3-glucanase of *Flavobacterium*. A black box indicates identical amino acids, and a gray box indicates amino acids conserved in at least two of the three sequences. Abbreviations: EK-14, Agl-EK14 of *Flavobacterium* sp. EK-14; anhuiense, putative α-1,3-glucanase of *F. anhuiense* M168 (WP_119790483.1); johnsoniae, putative α-1,3-glucanase of *F. johnsoniae* UW101 (ABQ06220.1).

(A)

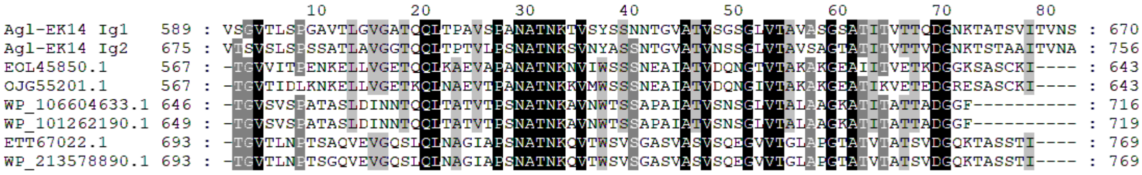

(B)

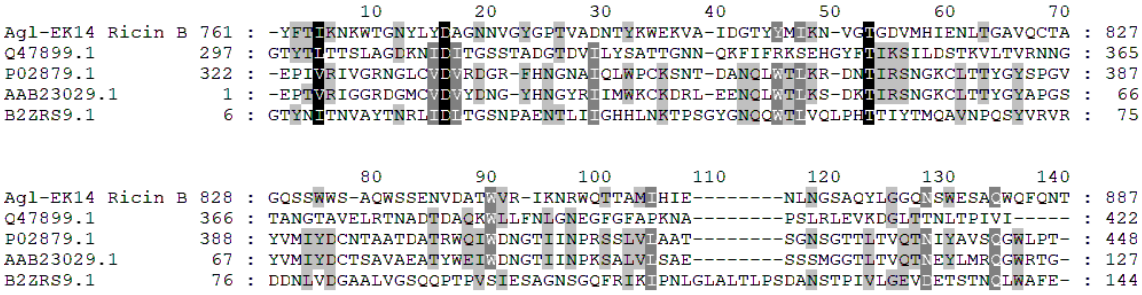

Supplementary Fig. S3. Multiple alignments of amino acid sequences of the immunoglobulin-like domain of Agl-EK14 with other enzymes (A) and Ricin B of Agl-EK14 with other enzymes (B). Amino acid sequences were aligned by ClustalW. The residue numbers of the first and last amino acids in each line are shown on the left and right. (A) A black box indicates completely identical amino acids. Amino acids conserved in at least six of the seven sequences are indicated by a gray box. Amino acids conserved in at least five of the seven sequences are indicated by a light gray box. Agl-EK14 Ig1, Agl-EK14 of *Flavobacterium* sp. EK-14 (589–670 amino acids); Agl-EK14 Ig2, Agl-EK14 of *Flavobacterium* sp. EK-14 (675–756 amino acids); EOL45850.1, metalloprotease of *Enterococcus caccae* ATCC BAA-1240; OJG55201.1, metalloprotease of *Enterococcus haemoperoxidus*; WP_106604633.1, GH9 protein of *Labilibaculum filiforme*; ETT67022.1, GH family protein of *Paenibacillus* sp. FSL H8-457; WP_213578890.1, glycosylhydrolase of *Paenibacillus lautus.* (B) A black box indicates all similar amino acids. Amino acids similar to at least four of the five sequences are indicated by a gray box. Amino acids similar to at least three of the five sequences are indicated by a light gray box. Abbreviations: Agl-EK14 Ricin B, Agl-EK14 of *Flavobacterium* sp. EK-14; Q47899.1, flavastacin of *Flavobacterium meningoseticum;*

P02879.1, Ricin B chain lectin domain; AAB23029.1, abrin-a B-chain lectin domain of *Abrus precatorius*; B2ZRS9.1, CNL of *Clitocybe nebularis*.

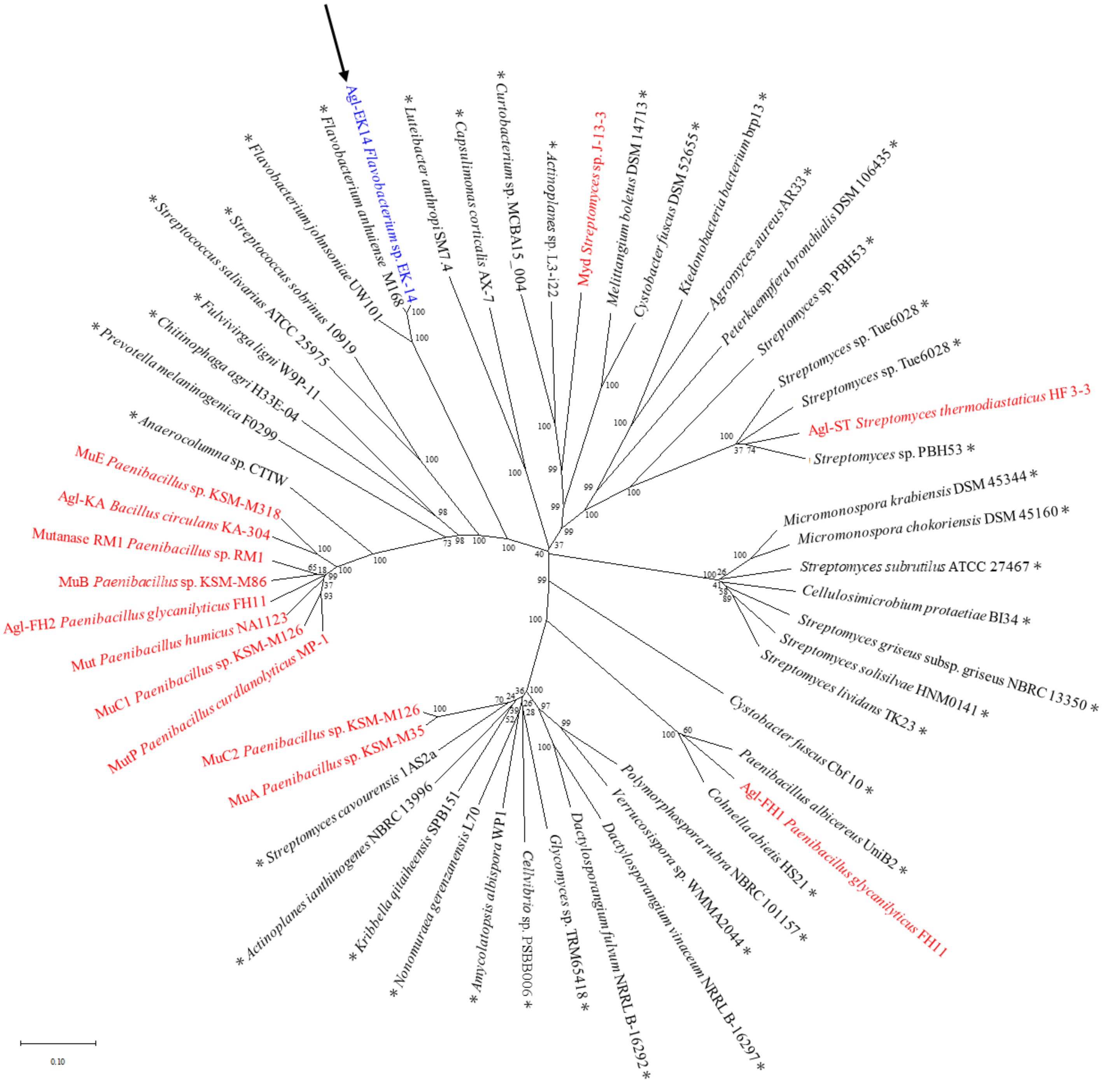

Supplementary Fig. S4. Phylogenetic analysis of the catalytic domains of Agl-EK14 and those of other GH87 α-1,3-glucanases and mycodextranases. The amino acid sequences of GH87 α-1,3-glucanases and mycodextranases were aligned by ClustalW, and the unrooted phylogenetic tree was constructed using the neighbor-joining method. The tree was drawn using MEGA11, and the numbers at the branches are bootstrap confidence percentages (%) based on 1000 resampled data sets. GenBank accession numbers of

GH87 α-1,3-glucanases and mycodextranases were given by the National Center for Biotechnology Information protein database. An arrow indicates Agl-EK14. Uncharacterized GH87 α-1,3-glucanases and mycodextranases indicated by asterisks are as follows: α-1,3-glucanase from *Capsulimonas corticalis* AX-7 (BDI29047.1), α-1,3-glucanase from *Luteibacter anthropi* SM7.4 (URX61610.1), α-1,3-glucanase from *Micromonospora chokoriensis* DSM 45160 (SCF30157.1), α-1,3-glucanase from *Micromonospora krabiensis DSM* 45344 (SBV25278.1), α-1,3-glucanase from *Streptomyces subrutilus* ATCC 27467 (QEU82241.1), α-1,3-glucanase from *Cellulosimicrobium protaetiae* BI34 (QJW35263.1), α-1,3-glucanase from *Streptomyces griseus* subsp. griseus NBRC 13350 (BAG23451.1), α-1,3-glucanase from *Streptomyces lividans* TK23 (BDE43507.1), α-1,3-glucanase from *Streptomyces solisilvae* HNM0141 (QPI55097.1), α-1,3-glucanase from *F. johnsoniae* UW101 (ABQ06220.1), α-1,3-glucanase from *F. anhuiense* M168 (WP_119790483.1), α-1,3-glucanase from *Streptococcus salivarius* ATCC 25975 (ARI60364.1), α-1,3-glucanase from *Streptococcus sobrinus* 10919 (AWN21666.1), α-1,3-glucanase from *Chitinophaga agri* H33E-04 (QHS58067.1), α-1,3-glucanase from *Fulvivirga ligni* W9P-11 (UII24169.1), α-1,3-glucanase from *Prevotella melaninogenica* F0299 (QUB68149.1), α-1,3-glucanase from *Anaerocolumna* sp. CTTW (BCJ98831.1), α-1,3-glucanase from *Cystobacter fuscus* Cbf 10 (WNG18114.1), α-1,3-glucanase from *Cohnella abietis* HS21 (BBI30922.1), α-1,3-glucanase from *Paenibacillus albicereus* UniB2 (QJC50342.1), α-1,3-glucanase from *Dactylosporangium fulvum* NRRL B-16292 (UWP84950.1), α-1,3-glucanase from *Dactylosporangium vinaceum* NRRL B-16297 (UAB99015.1), α-1,3-glucanase from *Polymorphospora rubra* NBRC 101157 (BCJ68800.1), α-1,3-glucanase from *Verrucosispora* sp. WMMA2044 (WBB48106.1), α-1,3-glucanase from *Kribbella qitaiheensis* SPB151 (QNE20272.1), α-1,3-glucanase from *Actinoplanes ianthinogenes* NBRC 13996 (BCJ42003.1), α-1,3-glucanase from *Streptomyces cavourensis* 1AS2a (ATY94307.1), α-1,3-glucanase from *Amycolatopsis albispora* WP1 (AXB48709.1), α-1,3-glucanase from *Nonomuraea gerenzanensis* L70 (UBU15042.1), α-1,3-glucanase from *Glycomyces* sp. TRM65418 (QZD55419.1), α-1,3-glucanase from *Cellvibrio* sp. PSBB006 (ARU29420.1), mycodextranase from *Streptomyces* sp. PBH53 (AKN70696.1), mycodextranase from *Streptomyces* sp. NRRL 30748 (ABC87502.1), mycodextranase from *Streptomyces* sp. Tue6028 (PBC59932.1), mycodextranase from

Amycolatopsis vastitatis (OXM62597.1), mycodextranase from *Peterkaempfera bronchialis* DSM 106435 (AXI76714.1), mycodextranase from *Agromyces aureus* AR33 (ANJ26885.1), mycodextranase from *Ktedonobacteria bacterium* brp13 (BCL81308.1), mycodextranase from *Melittangium boletus* DSM 14713 (ATB32882.1), mycodextranase from *Cystobacter fuscus* DSM 52655 (ATB43383.1), mycodextranase from *Curtobacterium* sp. MCBA15_004 (WIA96983.1), and mycodextranase from *Actinoplanes* sp. L3-i22 (BCY08438.1). Characterized GH87 α-1,3-glucanases and mycodextranases are as follows: Agl-KA from *B. circulans* KA-304 (BAE98302.1), MuE from *Paenibacillus* sp. KSM-M318 (BAH10514.1), mutanase RM1 from *Paenibacillus* sp. RM1 (E16590), MuB from *Paenibacillus* sp. KSM-M86 (BAF56208.1), Mut from *Paenibacillus humicus* NA1123 (BAI23187.1), Agl-FH2 from *P. glycanilyticus* FH11 (BAP10900.2), MuC1 from *Paenibacillus* sp. KSM-M126 (BAG15879.1), MutP from *P. curdlanolyticus* MP-1 (ADT91063.1), Agl-FH1 from *P. glycanilyticus* FH11 (BAP10900.1), MutC2 from *Paenibacillus* sp. KSM-M126 (BAG15880.1), MuA from *Paenibacillus* sp. KSM-M35 (BAG15878.1), Myd from *Streptomyces* sp. J-13-3 (BAB62749.1), and Agl-ST from *S. thermodiastaticus* HF 3-3 (BCK59654.1).

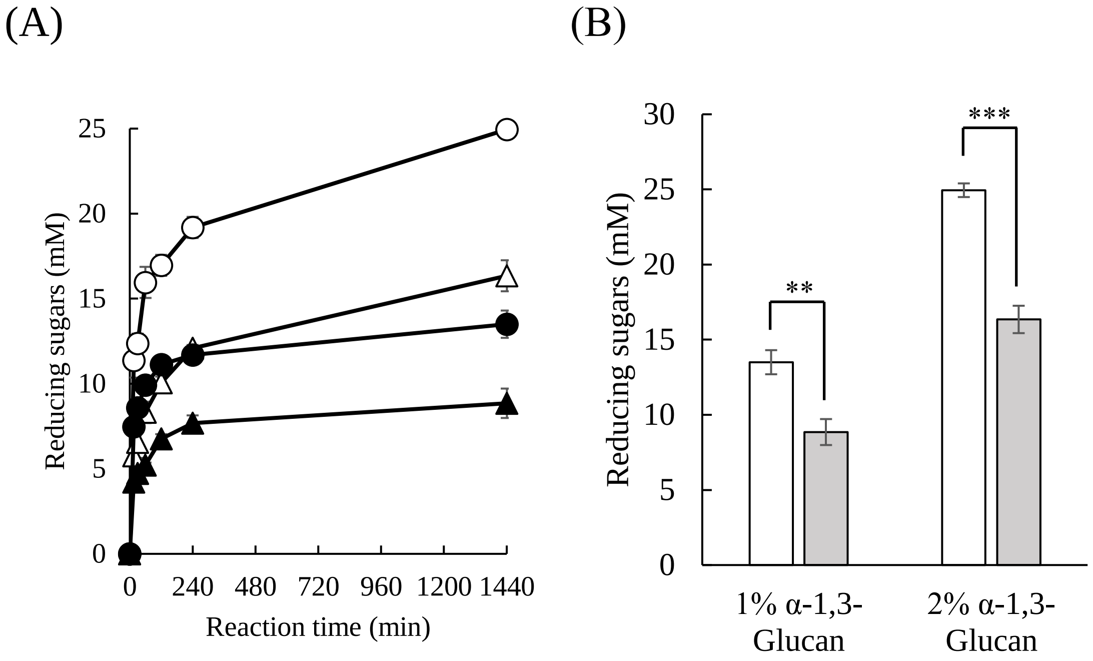

Supplementary Fig. S5. Comparison of α-1,3-glucan hydrolysis of Agl-EK14 with Agl-KA (A), and the amounts of reducing sugars released by Agl-EK14 and Agl-KA after 24 h of reaction (B). The reaction mixtures containing 1.0 nmol/ml α-1,3-glucanase, 50 mM potassium phosphate (pH 6.5), and α-1,3-glucan were incubated at 37°C. (A) Each data point represents the mean ± SD from triplicate experiments. Symbols: solid circles, 1% α-1,3-glucan hydrolyzed by Agl-EK14; open circles, 2% α-1,3-glucan hydrolyzed by Agl-EK14; solid triangles, 1% α-1,3-glucan hydrolyzed by Agl-KA; open triangles, 2% α-1,3-glucan hydrolyzed by Agl-KA. (B) White bars, Agl-EK14; gray bars, Agl-KA. The values are the means ± SD from triplicate experiments. Student's *t*-test was used to compare the amounts of reducing sugar released by Agl-EK14 and Agl-KA. Asterisks indicate significant differences (**$p < 0.01$, ***$p < 0.001$).

Peak 2

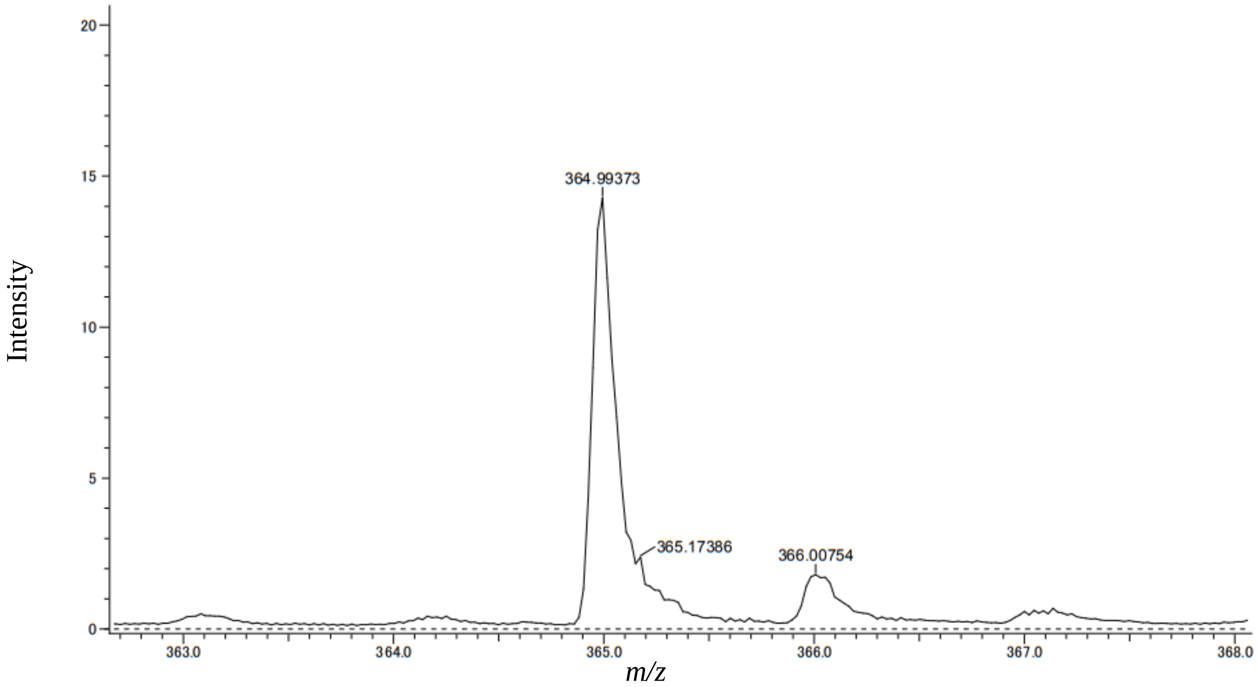

364.99373
365.17386
366.00754
Intensity
m/z

Peak 3

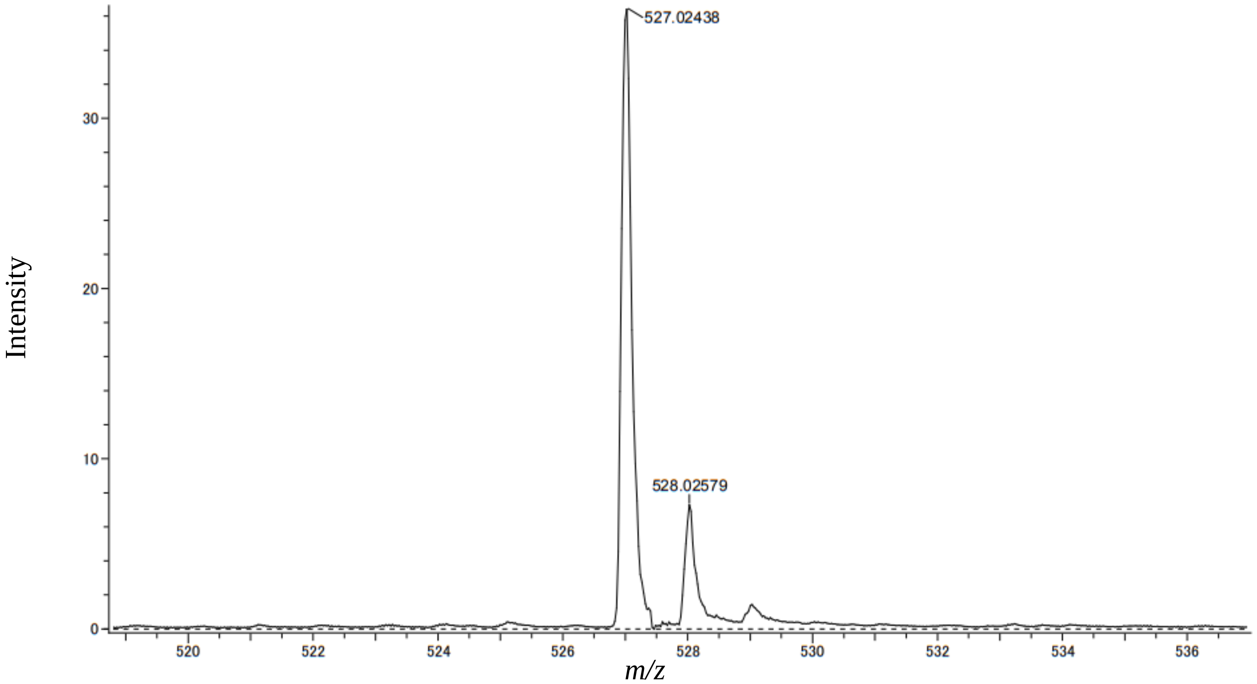

527.02438
528.02579
Intensity
m/z

Peak 4

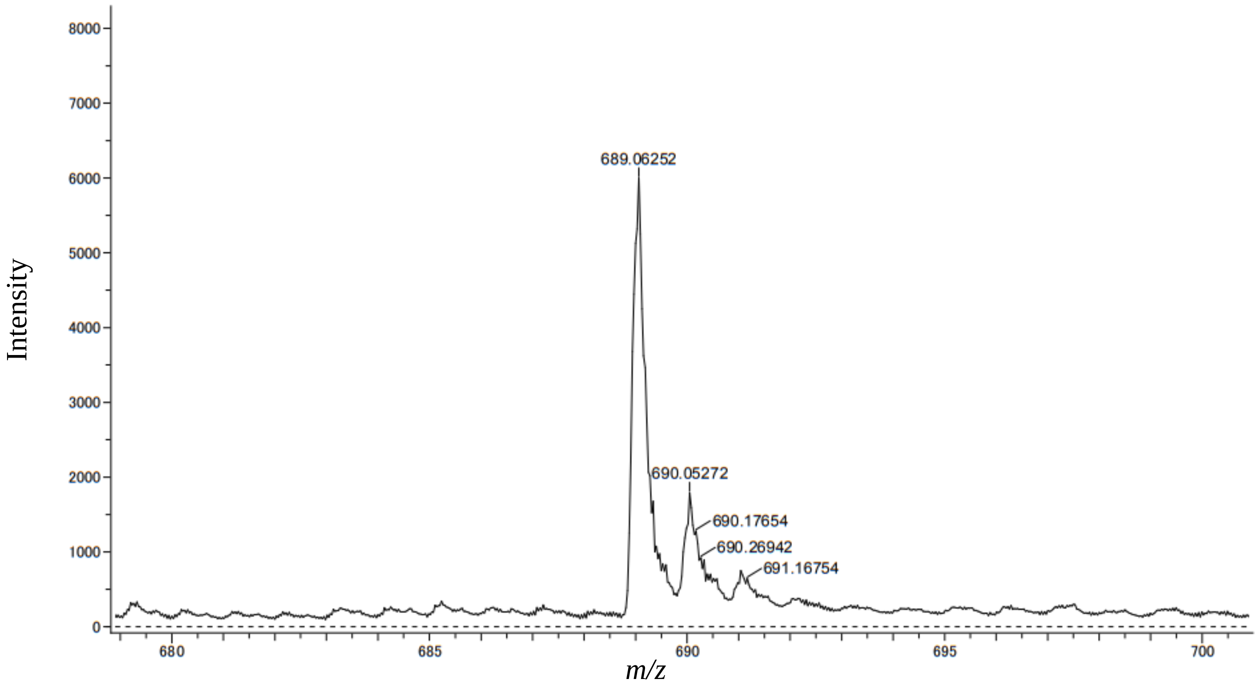

Supplementary Fig. S6. ESI-MS analysis of oligosaccharides in peaks 2 to 4 separated by HPLC. After separation by HPLC with an amide column, peaks 2 to 4 were collected, and collected samples were directly injected into the mass spectrometer (AccuTOF™ JMS-T100 LC, Jeol, Japan).

Light image BGBD-GFP AGBD-RFP

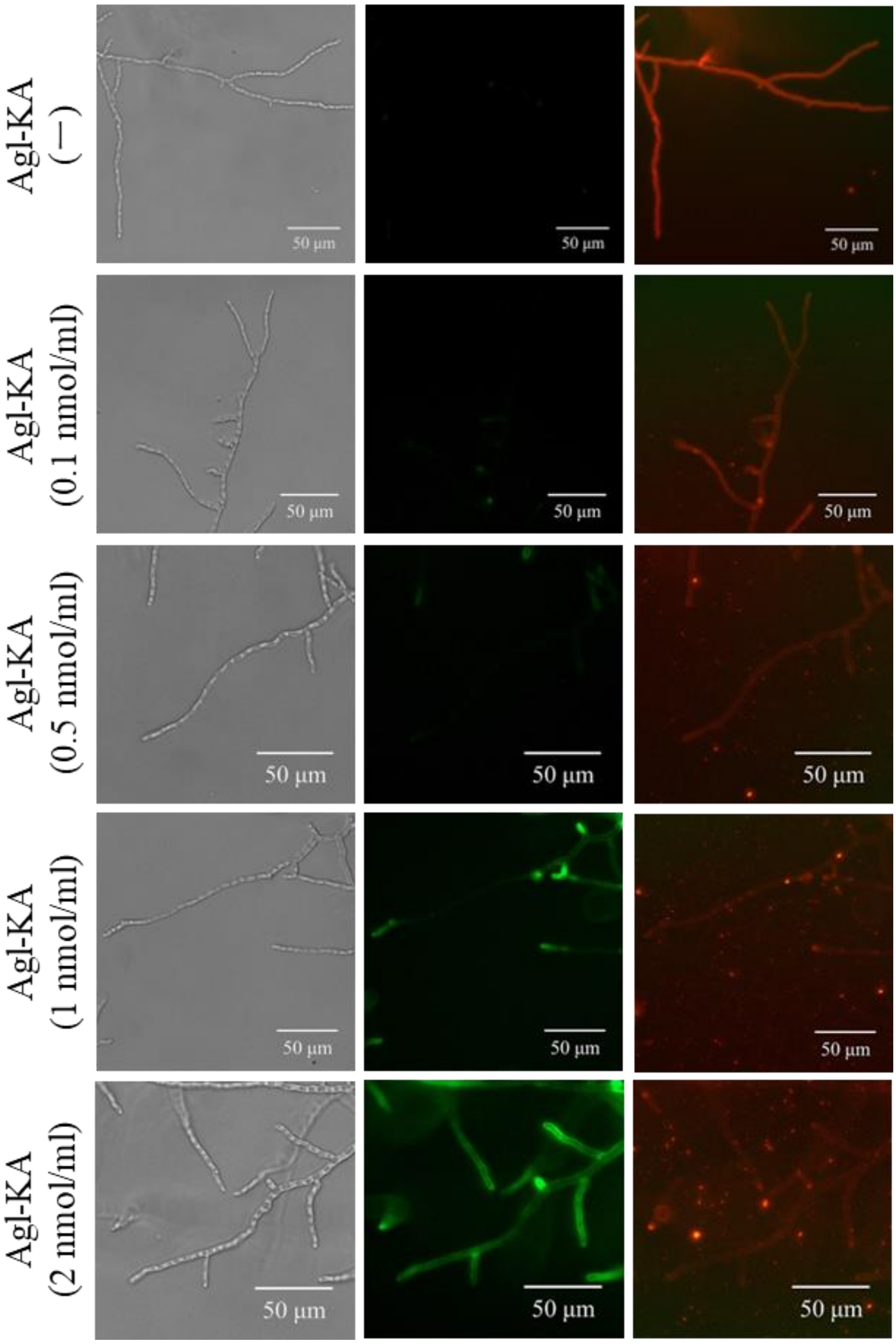

Supplementary Fig. S7. Cell wall binding assay of BGBD-GFP and AGBD-RFP for *A. oryzae* live mycelia. *A. oryzae* live mycelia were treated with Agl-KA at 0–2 nmol/ml. After 30-h treatment, mycelia were stained with AGBD-RFP (1.0 nmol/ml) and BGBD-GFP (1.0 nmol/ml).

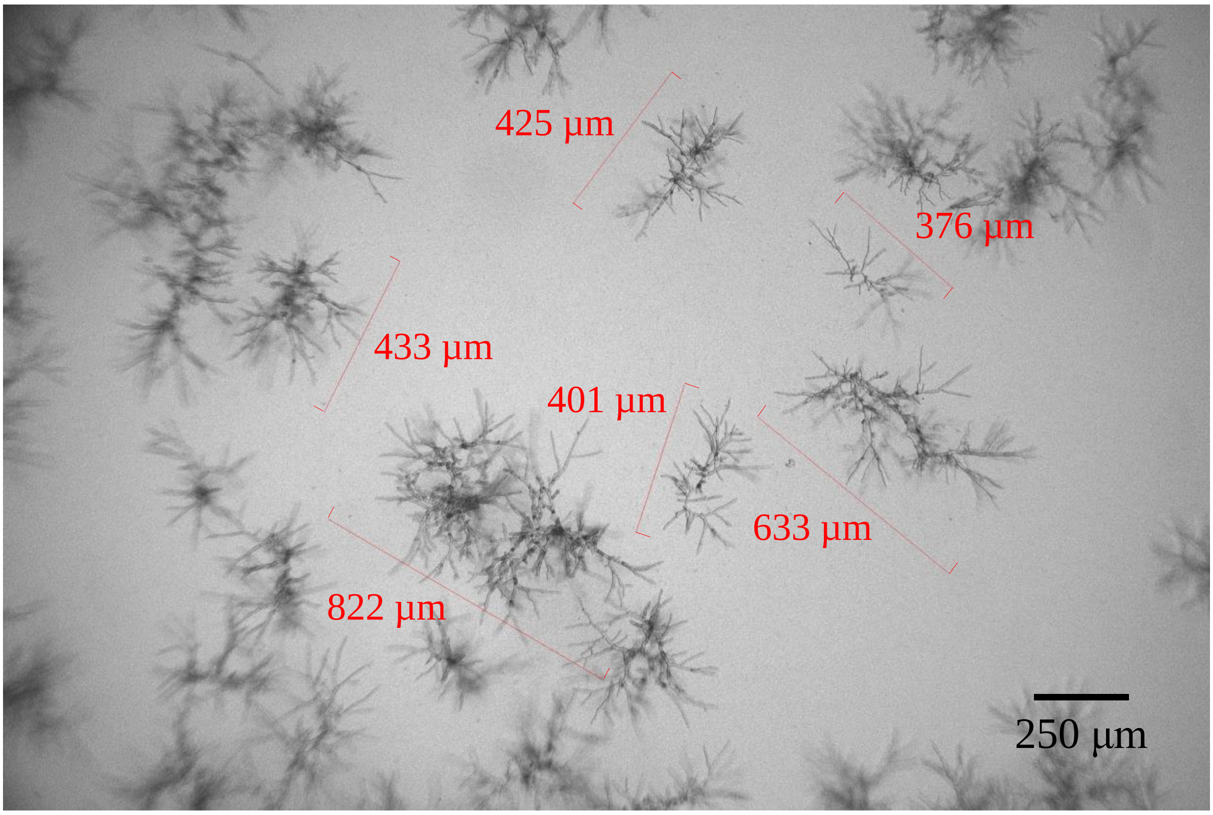

Supplementary Fig. S8. The diameters of colonies formed by *Aspergillus oryzae* treated with Agl-EK14, Chi19MK, and BgluC16MK. The colony diameters were measured for several colonies located in the center of the lower right photograph in Fig. 7. The software “MicroStudio” (WRAYMER INC.; Osaka, Japan) was used for size measurement.

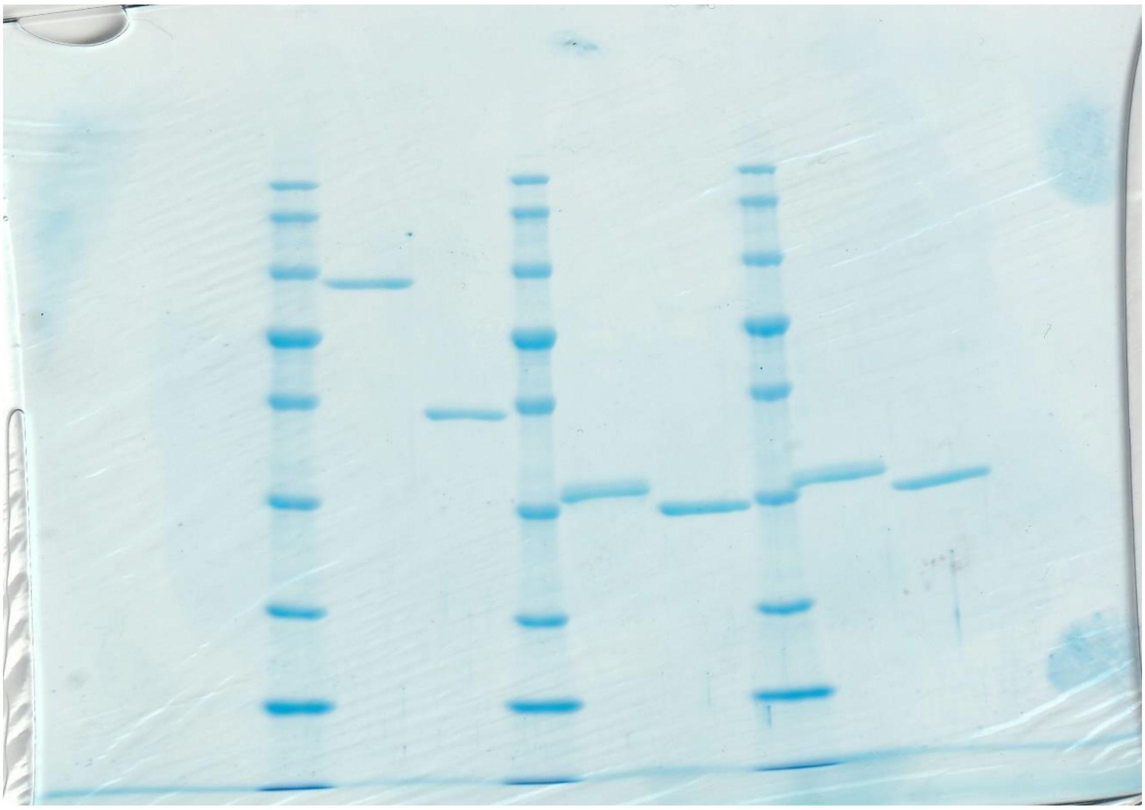

Supplementary Fig. S9. The original image of the SDS-PAGE analysis of the purified proteins used to construct Figure 1B.
